# Supplementary material for: In silico assessment of arrhythmic risk following the implantation of engineered heart tissues in porcine hearts with varying infarct locations
Source: PLoS Comput Biol. 2026 Apr 3;22(4):e1013740. doi: 10.1371/journal.pcbi.1013740 (PMC13108890; doi:10.1371/journal.pcbi.1013740)
Supplement: S1 Section — (PDF) [file pcbi.1013740.s001.pdf]

S1 Section. Delays of anterograde-retrograde propagation at the PMJ

As shown in S1 Fig, both anterograde and retrograde electrical propagation across the PMJs was successfully captured. To quantify direction-dependent propagation delays, a localized region of the CS-BiV complex near pacing site 15 (pig 6) was analyzed following the final S1 stimulus. The propagation delay at a PMJ was defined as the mean activation time difference between the CS endpoint and its associated myocardial nodes. As defined in Section 2.1, these myocardial nodes were identified as ventricular nodes within a 0.5 mm radius of the CS endpoint. Across the full CS-BiV domain, complex phenomena can introduce confounding effects that preclude a clean calculation. These include the simultaneous depolarization of a PMJ from both the CS and myocardial tissue, as well as variations in local PMJ density that alter the source–sink mismatch and modify conduction delays by changing electrotonic loading effects [1]. Consequently, the analysis was restricted to a reduced postero-apical region near the pacing site, focusing specifically on the three PMJ propagation events highlighted in S1 Fig.

As illustrated in S1 Fig, three epicardial breakthroughs are observed: one (red star in S1 Fig) originating from stimulation at endocardial pacing site 15, producing a retrograde reentry, and two (black stars in S1 Fig) resulting from anterograde depolarization through distinct PMJs. The retrograde delay was zero, suggesting that the activation of the CS endpoint occurred simultaneously with that of the connected myocardial nodes. However, this measurement was limited by the temporal resolution of our simulations (1 ms). For anterograde conduction, a mean delay of 1.1 ms was measured across the two PMJs propagating in the anterograde direction.

Computational studies of bidirectional conduction have calibrated higher delay values, particularly in human-oriented modeling and simulation, largely based on canine experimental data. For example, Gillette et al. [2] used anterograde and retrograde propagation delays of 8 and 3 ms, respectively. In closer agreement with the present work, Riebel et al. [3] used delays of 2 and 1 ms for anterograde and retrograde propagation. Experimentally, Veenstra et al. [4] reported anterograde delays of 2–4 ms in canine studies and reviewed evidence suggesting that anterograde delays below 5 ms are more physiological than larger values. Retrograde delays are consistently reported to be shorter than anterograde delays, reflecting faster myocardium-to-CS conduction, likely due to the greater electrotonic load imposed on the CS endpoint by the bulkier myocardial tissue, a source–sink mismatch widely observed *in silico* [5, 6, 7]. We previously showed that this effect is accentuated in pigs [7], as PMJs are located deeper within the myocardium than in dogs and humans and contact a larger myocardial mass for a given PMJ size [8, 9], thereby supporting the lower retrograde delay values obtained in the present study. Under this hypothesis, larger anterograde delays than those reported experimentally in dogs would also be expected. Indeed, when myocardial nodes located farther from the CS endpoint are considered in the anterograde delay calculation, higher values are obtained (mean anterograde delays of 1.7 and 2.7 ms for myocardial nodes located up to 0.6 and 1 mm from the CS endpoint, respectively). This observation is particularly relevant given that the experimental measurements used were inherently limited in spatial resolution and are typically unable to resolve distances smaller than 1 mm [4].

Taken together, we contend that the anterograde and retrograde delays employed here are consistent with physiological values: anterograde delays are systematically greater than retrograde delays, and the retrograde delay can reasonably be assumed to be zero due to the increased source–sink mismatch associated with the deeper CS in pigs. Future studies may further refine these parameters by incorporating porcine-specific experimental data, which are currently lacking.

References

[1] Vigmond EJ, Clements C. Construction of a computer model to investigate sawtooth effects in the Purkinje system. IEEE Transactions on Biomedical Engineering. 2007;54:389-99. doi:10.1109/TBME.2006.888817.

[2] Gillette K, Gsell MAF, Bouyssier J, Prassl AJ, Neic A, Vigmond EJ, et al. Automated framework for the inclusion of a His–Purkinje system in cardiac digital twins of ventricular electrophysiology. Annals of Biomedical Engineering. 2021;49:3143-53. doi:10.1007/s10439-021-02825-9.

[3] Riebel LL, Wang ZJ, Martinez-Navarro H, Trovato C, Camps J, Berg LA, et al. *In silico* evaluation of cell therapy in acute versus chronic infarction: Role of automaticity, heterogeneity and Purkinje in human. Scientific Reports. 2024;14:21584. doi:10.1038/s41598-024-67951-5.

[4] Veenstra RD, Joyner RW, Rawling DA. Purkinje and ventricular activation sequences of canine papillary muscle. Effects of quinidine and calcium on the Purkinje-ventricular conduction delay. Circulation Research. 1984;54:500-15. doi:10.1161/01.RES.54.5.500.

[5] Berenfeld O, Jalife J. Purkinje-muscle reentry as a mechanism of polymorphic ventricular arrhythmias in a 3-dimensional model of the ventricles. Circulation Research. 1998;82:1063-77. doi:10.1161/01.RES.82.10.1063.

[6] Behradfar E, Nygren A, Vigmond EJ. The role of Purkinje-myocardial coupling during ventricular arrhythmia: A modeling study. PLoS ONE. 2014;9:e88000. doi:10.1371/journal.pone.0088000.

[7] Rosales RM, Wu M, Claus P, Janssens S, Ríos-Muñoz GR, Fernández-Santos ME, et al. Integrated multi-modal data analysis for computational modeling of healthy and location-dependent myocardial infarction conditions in porcine hearts [Preprint]. bioRxiv; 2025 [cited 2025 November 5]. Available from: `https://doi.org/10.1101/2025.10.31.685788`.

[8] Lelovas PP, Kostomitsopoulos NG, Xanthos TT. A comparative anatomic and physiologic overview of the porcine heart. Journal of the American Association for Laboratory Animal Science. 2014;53:432-8. Available from: `https://pmc.ncbi.nlm.nih.gov/articles/PMC4181683/`.

[9] Garcia-Bustos V, Sebastian R, Izquierdo M, Rios-Navarro C, Bodí V, Chorro FJ, et al. Changes in the spatial distribution of the Purkinje network after acute myocardial infarction in the pig. PLoS ONE. 2019;14:e0212096. doi:10.1371/journal.pone.0212096.
