# Supplementary figures and images for: In silico assessment of arrhythmic risk following the implantation of engineered heart tissues in porcine hearts with varying infarct locations
Source: PLoS Comput Biol. 2026 Apr 3;22(4):e1013740. doi: 10.1371/journal.pcbi.1013740 (PMC13108890; doi:10.1371/journal.pcbi.1013740)

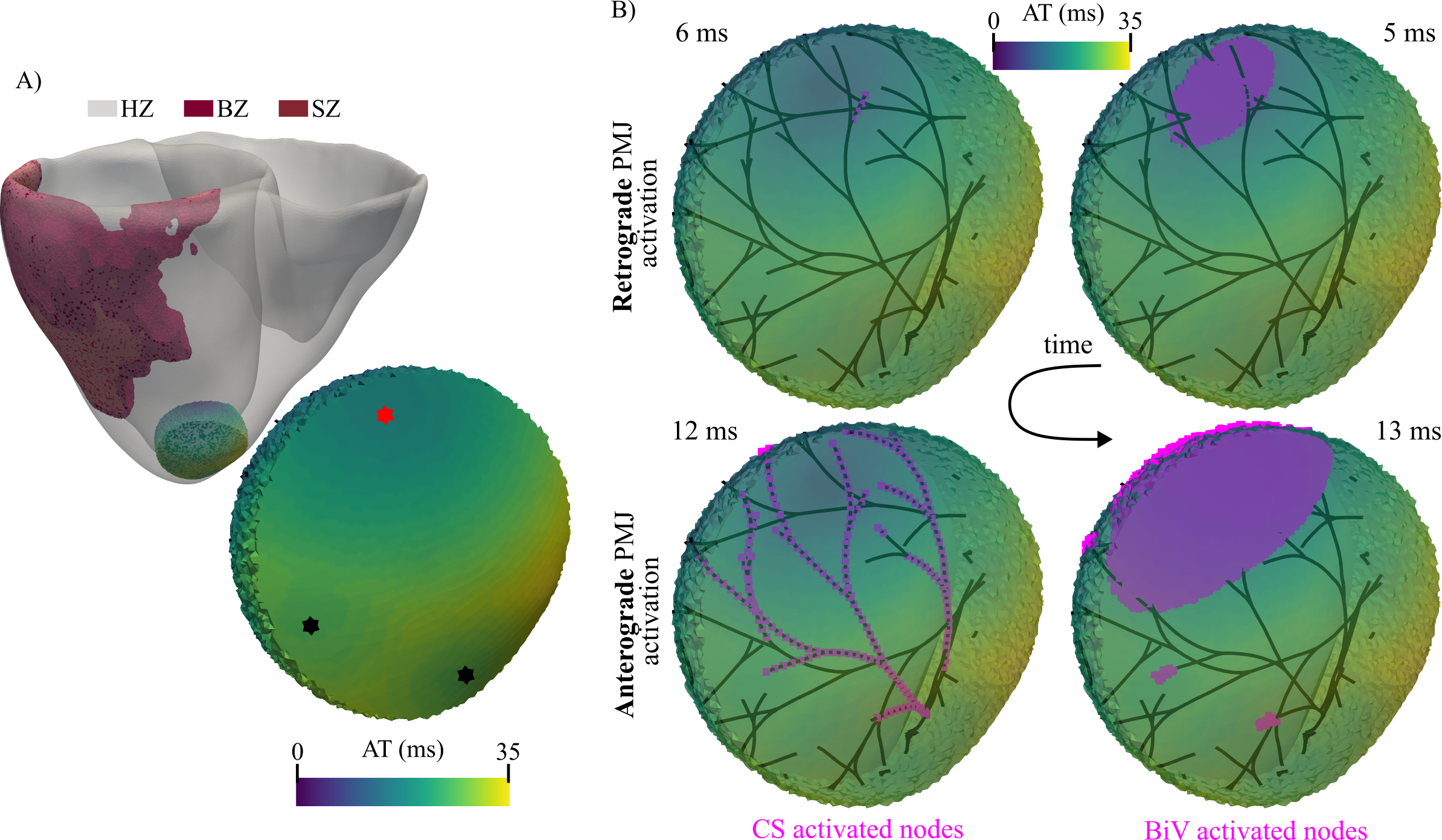

Supplement: S1 Fig — A- Analysis of the postero-apical region (pig 6) following the last S1 stimulus at pacing site 15. Three distinct epicardial breakthroughs are observed, arising from the interaction between endocardial stimulation and CS depolarization. B- The endocardial stimulus applied at 0 ms (snapshot at 5 ms, top-right) triggers retrograde propagation at a PMJ (CS node ID 2350) at 6 ms (top-left), producing the first epicardial breakthrough (red star, panel A). This wavefront propagates rapidly through the CS (12 ms, bottom-left), culminating in two anterograde PMJ activations at 13 ms (bottom-right, CS node IDs 1592 and 17346), which manifest as the two secondary breakthroughs (black stars, panel A). (TIFF) [file pcbi.1013740.s002.tiff]

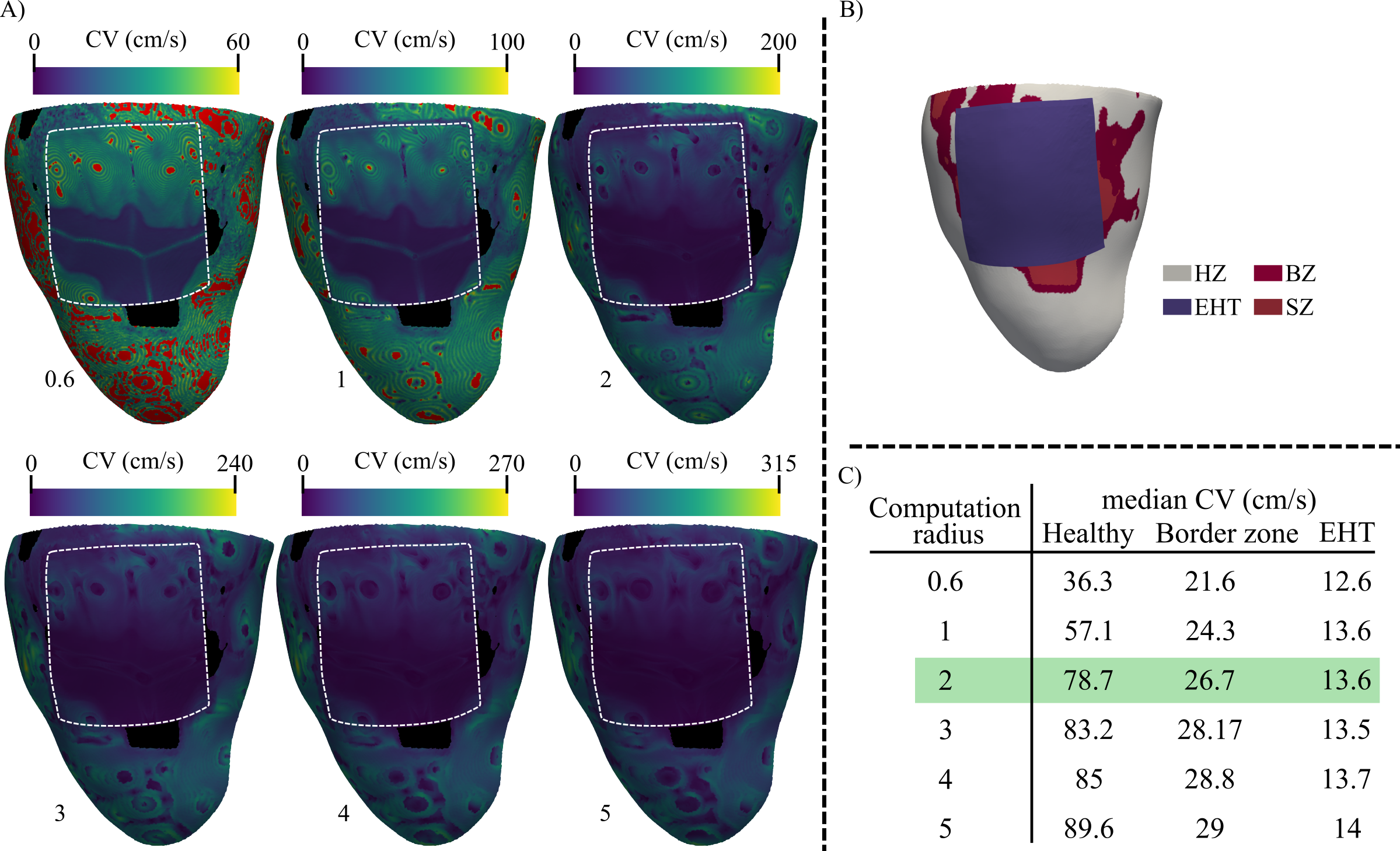

Supplement: S2 Fig — A- Node-wise CV computation was performed on the lateral surface of pig 6 following the last S1 stimulus at pacing site 15. The CV magnitude for a given node was calculated as the mean ratio of Euclidean distance to activation time difference relative to all neighboring nodes within a specified search radius. Sensitivity analysis of the search radius revealed that smaller radii yielded numerical artifacts (infinite values, shown in red) due to neighboring nodes sharing identical activation times (1 ms resolution). CV values in the fast-conducting healthy zone (HZ) stabilized as the radius increased. B- Anatomical segmentation of the lateral ventricular face, highlighting HZ, border zone (BZ), scar zone (SZ), and engineered heart tissue (EHT) regions. C- Median CV values calculated for the HZ, BZ, and EHT across the different search radii tested. A trade-off radius of 2 mm was selected for all subsequent calculations (highlighted in green), as it represented the minimum distance required to ensure numerical stability and convergence across all tissue zones. (TIFF) [file pcbi.1013740.s003.tiff]
