# Supplementary material for: In silico assessment of arrhythmic risk following the implantation of engineered heart tissues in porcine hearts with varying infarct locations
Source: PLoS Comput Biol. 2026 Apr 3;22(4):e1013740. doi: 10.1371/journal.pcbi.1013740 (PMC13108890; doi:10.1371/journal.pcbi.1013740)
Supplement: S1 Table — G1 simulations correspond to the baseline MI-related evaluation. LCx pigs (4–7) are shown at the left table, and LAD pigs (8–12) are shown at the right table. PS: pacing site, NC: no capture, NR: no reentry, nsVT: non-sustained VT, sVT: sustained VT. (PDF) [file pcbi.1013740.s004.pdf]

S1 Table. Results of the arrhythmia inducibility protocol obtained in the G1 simulations. G1 simulations correspond to the baseline MI-related evaluation. LCx pigs (4-7) are shown at the left table, and LAD pigs (8-12) are shown at the right table. PS: pacing site, NC: no capture, NR: no reentry, nsVT: non-sustained VT, sVT: sustained VT.

**Pig**	**PS**	**S2** (ms)			
		250	265	280	295
**4**	4	NC	NR	NR	NR
	5	NC	NC	NC	NR
	1	NC	NR	NR	NR
	11	NC	NC	NC	NC
	10	NC	NC	NR	NR
	13	NC	NC	NR	NR
	17	NC	NR	NR	NR
**5**	4	NC	NC	nsVT	nsVT
	6	NC	nsVT	nsVT	nsVT
	12	NC	NC	nsVT	nsVT
	11	NC	NC	nsVT	nsVT
	15	NC	NC	NC	NC
	16	NC	nsVT	nsVT	nsVT
	17	NC	nsVT	nsVT	nsVT
**6**	4	NC	NC	NC	NR
	5	NC	NC	NC	NC
	6	NC	NR	NR	NR
	15	NC	NR	NR	NR
	16	NC	NR	NR	NR
	17	NC	NR	NR	NR
**7**	4	NC	NC	nsVT	nsVT
	6	NC	NC	NC	nsVT
	15	NC	NC	NC	NC
	16	NC	nsVT	nsVT	nsVT
	17	NC	nsVT	nsVT	nsVT

**Pig**	**PS**	**S2** (ms)			
		250	265	280	295
**8**	2	NC	nsVT	nsVT	NR
	3	NC	sVT	NR	NR
	12	NC	NC	nsVT	nsVT
	7	NC	NC	NC	NC
	15	NC	NC	NC	NC
	18	NC	NC	NC	nsVT
**9**	3	NC	NC	nsVT	NR
	7	NC	NC	nsVT	nsVT
	10	NC	NC	nsVT	sVT
	16	NC	NC	nsVT	nsVT
	18	NC	NC	NC	NC
**10**	3	NC	NC	nsVT	NR
	8	NC	NC	NC	NC
	10	NC	NC	sVT	sVT
	16	NC	NC	NR	NR
	18	NC	NC	sVT	sVT
**11**	2	NC	nsVT	nsVT	nsVT
	12	NC	NC	sVT	sVT
	7	NC	NC	sVT	sVT
	9	NC	NC	NC	NC
	17	NC	NC	NC	NC
	18	NC	NC	sVT	nsVT
**12**	2	NC	NR	NR	NR
	3	NC	NR	NR	NR
	12	NC	NC	NR	sVT
	7	NC	NC	NR	NR
	11	NC	NC	NR	NR
	18	NC	NC	NC	nsVT
