# Supplementary material for: In silico assessment of arrhythmic risk following the implantation of engineered heart tissues in porcine hearts with varying infarct locations
Source: PLoS Comput Biol. 2026 Apr 3;22(4):e1013740. doi: 10.1371/journal.pcbi.1013740 (PMC13108890; doi:10.1371/journal.pcbi.1013740)
Supplement: S2 Table — G2 simulations correspond to the assessment of the CS presence. LCx pigs (6–7) are shown at the top of the table, and LAD pigs (11–12) are shown at the bottom of the table. PS: pacing site, NC: no capture, NR: no reentry, nsVT: non-sustained VT, sVT: sustained VT. Note that results in column “with CS” are identical to the outcomes of G1 simulations (S1 Table) where an S2 interval of 295 ms was employed. Additionally, pacing sites which resulted in NC in the G1 simulations were excluded (-). (PDF) [file pcbi.1013740.s005.pdf]

**S2 Table. Results of the arrhythmia inducibility protocol obtained in the G2 simulations.** G2 simulations correspond to the assessment of the CS presence. LCx pigs (6-7) are shown at the top of the table, and LAD pigs (11-12) are shown at the bottom of the table. PS: pacing site, NC: no capture, NR: no reentry, nsVT: non-sustained VT, sVT: sustained VT. Note that results in column "with CS" are identical to the outcomes of G1 simulations (S1 Table) where an S2 interval of 295 ms was employed. Additionally, pacing sites which resulted in NC in the G1 simulations were excluded (-).

| **Pig** | **PS** | **S2 295** (ms) | |
|---|---|---|---|
| | | with CS | without CS |
| **6** | 4 | NR | NC |
| | 5 | NC | - |
| | 6 | NR | sVT |
| | 15 | NR | NR |
| | 16 | NR | NR |
| | 17 | NR | NR |
| **7** | 4 | nsVT | nsVT |
| | 6 | nsVT | NC |
| | 15 | NC | - |
| | 16 | nsVT | NR |
| | 17 | nsVT | NR |
| **11** | 2 | nsVT | nsVT |
| | 12 | sVT | nsVT |
| | 7 | sVT | nsVT |
| | 9 | NC | - |
| | 17 | NC | - |
| | 18 | nsVT | nsVT |
| **12** | 2 | NR | nsVT |
| | 3 | NR | sVT |
| | 12 | sVT | nsVT |
| | 7 | NR | nsVT |
| | 11 | NR | nsVT |
| | 18 | nsVT | nsVT |
