# Supplementary material for: In silico assessment of arrhythmic risk following the implantation of engineered heart tissues in porcine hearts with varying infarct locations
Source: PLoS Comput Biol. 2026 Apr 3;22(4):e1013740. doi: 10.1371/journal.pcbi.1013740 (PMC13108890; doi:10.1371/journal.pcbi.1013740)
Supplement: S3 Table — G3 simulations correspond to the assessment of the remuscularization and the location of the EHT implantation. LCx pigs (4–7) are shown at the left table, and LAD pigs (8–12) are shown at the right table. PS: pacing site, MI: baseline pre-EHT, L1: EHT at L1, L2: EHT at L2, NC: no capture, NR: no reentry, nsVT: non-sustained VT, sVT: sustained VT. Note that results in column “MI” are identical to the outcomes of G1 simulations (S1 Table) where an S2 interval of 295 ms was employed. Additionally, a low EHT conductivity was used, and pacing sites that resulted in NC in the G1 simulations were excluded (-). (PDF) [file pcbi.1013740.s006.pdf]

S3 Table. Results of the arrhythmia inducibility protocol obtained in the G3 simulations. G3 simulations correspond to the assessment of the remuscularization and the location of the EHT implantation. LCx pigs (4-7) are shown at the left table, and LAD pigs (8-12) are shown at the right table. PS: pacing site, MI: baseline pre-EHT, L1: EHT at L1, L2: EHT at L2, NC: no capture, NR: no reentry, nsVT: non-sustained VT, sVT: sustained VT. Note that results in column "MI" are identical to the outcomes of G1 simulations (S1 Table) where an S2 interval of 295 ms was employed. Additionally, a low EHT conductivity was used, and pacing sites that resulted in NC in the G1 simulations were excluded (-).

Pig	PS	**S2 295** (ms)		
		MI	L1	L2
**4**	4	NR	nsVT	NR
	5	NR	nsVT	nsVT
	1	NR	nsVT	nsVT
	11	NC	-	-
	10	NR	nsVT	nsVT
	13	NR	nsVT	NR
	17	NR	nsVT	NR
**5**	4	nsVT	nsVT	nsVT
	6	nsVT	nsVT	nsVT
	12	nsVT	nsVT	nsVT
	11	nsVT	nsVT	nsVT
	15	NC	-	-
	16	nsVT	nsVT	nsVT
	17	nsVT	nsVT	nsVT
**6**	4	NR	sVT	sVT
	5	NC	-	-
	6	NR	sVT	nsVT
	15	NR	sVT	sVT
	16	NR	sVT	nsVT
	17	NR	sVT	nsVT
**7**	4	nsVT	nsVT	nsVT
	6	nsVT	nsVT	nsVT
	15	NC	-	-
	16	nsVT	nsVT	nsVT
	17	nsVT	nsVT	nsVT

Pig	PS	**S2 295** (ms)		
		MI	L1	L2
**8**	2	NR	nsVT	nsVT
	3	NR	NR	nsVT
	12	nsVT	nsVT	nsVT
	7	NC	-	-
	15	NC	-	-
	18	nsVT	nsVT	nsVT
**9**	3	NR	NR	NR
	7	nsVT	nsVT	nsVT
	10	sVT	nsVT	sVT
	16	nsVT	nsVT	nsVT
	18	NC	-	-
**10**	3	NR	NR	NR
	8	NC	-	-
	10	sVT	sVT	sVT
	16	NR	NR	NR
	18	sVT	nsVT	sVT
**11**	2	nsVT	sVT	nsVT
	12	sVT	sVT	sVT
	7	sVT	sVT	sVT
	9	NC	-	-
	17	NC	-	-
	18	nsVT	nsVT	nsVT
**12**	2	NR	NR	NR
	3	NR	NR	NR
	12	sVT	nsVT	NR
	7	NR	NR	nsVT
	11	NR	NR	NR
	18	nsVT	nsVT	nsVT
