# Supplementary material for: In silico assessment of arrhythmic risk following the implantation of engineered heart tissues in porcine hearts with varying infarct locations
Source: PLoS Comput Biol. 2026 Apr 3;22(4):e1013740. doi: 10.1371/journal.pcbi.1013740 (PMC13108890; doi:10.1371/journal.pcbi.1013740)
Supplement: S4 Table — G4 simulations correspond to the assessment of the EHT conductivity variation when the EHT was implanted in different locations. The left table correspond to the EHT location L1 and the right table correspond to the EHT location L2. LCx pigs (4–7) are shown at the top of the tables, and LAD pigs (8–12) are shown at the bottom of the tables. PS: pacing site, NR: no reentry, nsVT: non-sustained VT, sVT: sustained VT, C10/90: EHT with a conductivity equal to the 10/90% of the value found in the healthy adult-like tissue. Note that results in column “C10” are identical to the outcomes of G3 simulations (S3 Table) where an S2 interval of 295 ms and low EHT conductivity were employed. (PDF) [file pcbi.1013740.s007.pdf]

S4 Table. Results of the arrhythmia inducibility protocol obtained in the G4 simulations. G4 simulations correspond to the assessment of the EHT conductivity variation when the EHT was implanted in different locations. The left table correspond to the EHT location L1 and the right table correspond to the EHT location L2. LCx pigs (4-7) are shown at the top of the tables, and LAD pigs (8-12) are shown at the bottom of the tables. PS: pacing site, NR: no reentry, nsVT: non-sustained VT, sVT: sustained VT, C10/90: EHT with a conductivity equal to the 10/90% of the value found in the healthy adult-like tissue. Note that results in column "C10" are identical to the outcomes of G3 simulations (S3 Table) where an S2 interval of 295 ms and low EHT conductivity were employed.

**Pig**	**PS**	**S2 295** (ms)	
		C10	C90
**4**	1	nsVT	NR
**5**	6	nsVT	nsVT
**6**	15	sVT	nsVT
**7**	17	nsVT	nsVT
**8**	2	nsVT	nsVT
**9**	10	nsVT	nsVT
**10**	10	sVT	sVT
**11**	2	sVT	sVT
**12**	12	nsVT	nsVT

**Pig**	**PS**	**S2 295** (ms)	
		C10	C90
**4**	1	nsVT	NR
**5**	6	nsVT	nsVT
**6**	15	sVT	nsVT
**7**	17	nsVT	nsVT
**8**	2	nsVT	nsVT
**9**	10	sVT	sVT
**10**	10	sVT	sVT
**11**	2	nsVT	nsVT
**12**	12	NR	NR
