# Supplementary material for: In silico assessment of arrhythmic risk following the implantation of engineered heart tissues in porcine hearts with varying infarct locations
Source: PLoS Comput Biol. 2026 Apr 3;22(4):e1013740. doi: 10.1371/journal.pcbi.1013740 (PMC13108890; doi:10.1371/journal.pcbi.1013740)
Supplement: S5 Table — In the left table, the volumetric ratios between the MI and the BiV, as well as, between the BZ and the MI are presented. In the right table, the epicardial surface ratio of the SZ with whole MI is presented. LCx pigs (4–7) are depicted in the top of the tables while the LAD pigs (8–12) can be found at the bottom of the tables. (PDF) [file pcbi.1013740.s008.pdf]

S5 Table. Results of the characterization of the MI substrate. In the left table, the volumetric ratios between the MI and the BiV, as well as, between the BZ and the MI are presented. In the right table, the epicardial surface ratio of the SZ with whole MI is presented. LCx pigs (4-7) are depicted in the top of the tables while the LAD pigs (8-12) can be found at the bottom of the tables.

Pig	MI/BiV (%)	BZ/MI (%)
**4**	8.76	19.97
**5**	8.04	20.8
**6**	10.55	32.05
**7**	13.99	45.52
**8**	30.20	20.7
**9**	27.14	32.55
**10**	16.61	41.6
**11**	20.76	46.55
**12**	13.42	36.2

Pig	SZ/MI (%)
**4**	48.64
**5**	42.93
**6**	47.3
**7**	3.85
**8**	15.14
**9**	0.53
**10**	0
**11**	6.59
**12**	0
