# Supplementary material for: In silico assessment of arrhythmic risk following the implantation of engineered heart tissues in porcine hearts with varying infarct locations
Source: PLoS Comput Biol. 2026 Apr 3;22(4):e1013740. doi: 10.1371/journal.pcbi.1013740 (PMC13108890; doi:10.1371/journal.pcbi.1013740)
Supplement: Attachment — Submitted filename: Response_to_Reviewers.pdf [file pcbi.1013740.s014.pdf]

March 9, 2026

Re: Re-submission of manuscript "*In silico* assessment of arrhythmic risk following the implantation of engineered heart tissues in porcine hearts with varying infarct locations", with reference PCOMPBIOL-D-25-02377

Ricardo M. Rosales
University of Zaragoza
Zaragoza, Spain
rrosales@unizar.es

Editor in Chief
PLoS Computational Biology

Dear Editor,

We would like to thank you for the opportunity to revise our manuscript, "*In silico* assessment of arrhythmic risk following the implantation of engineered heart tissues in porcine hearts with varying infarct locations". We appreciate the careful review and the constructive suggestions provided by the Reviewers. We believe the manuscript has been substantially strengthened by incorporating these modifications.

The primary revisions are summarized below:

- *Refined Terminology and Context:* We have revised the manuscript to ensure technical precision and prevent misinterpretation. Specifically, we clarified the qualitative nature of our *in silico* versus experimental reentrant pattern comparisons, distinguished our arrhythmia inducibility protocol from previous work, and better defined our model calibration and how we represented engineered heart tissue (EHT) maturation. Furthermore, we highlighted the advances of our work within the context of novel modeling and simulation research, especially focused on EHT-mediated remuscularization therapy.
- *Enhanced Methodological Clarity:* The description of our methodology has been improved to be more self-contained, reducing the reliance on citations of previous work. We extended the descriptions of the optical mapping (OM) experiments, the *in silico* arrhythmia inducibility protocol, the modeling and functional parametrization of the conduction system (CS) and infarcted biventricular structures, pre- or post-implantation of the EHT. We also clarified the prior modeling validation against experimental data [1].
- Conductivity Modeling Assessment: We expanded the justification for our conductivity settings. To demonstrate the model's trustworthiness, we validated these settings by comparing *in silico* and experimental conduction velocities (CV). These computations are now detailed in a new section, "Simulated conduction velocities", addressing concerns regarding physiological realism in meshes with multiple edge-length resolutions.
- Improved CS-BiV Validation and Results: The characteristics and validation of the CS modeling are now explained in greater detail. The analysis of arrhythmogenicity in the presence or absence of the CS was significantly expanded with new text and figures, including transmembrane potential traces to reinforce our findings.
- Robustness and Uncertainty Analysis: To strengthen the model's foundation, we explicitly detailed the rationale behind parameter selection. We also extended the discussion on uncertainties and future directions, specifically regarding cross-species parametrization, CS functional/structural modeling, and simplifications in the modeling of EHT maturation and engraftment.
- Structural Refinements and Transparency: The manuscript has been slightly restructured for improved logical flow. We ensured full adherence to journal formatting guidelines and made all experimental data, simulation configuration files, and code openly available. Additionally, all figures were converted to high-resolution .tiff format, and a declaration of competing interests has been included.

Following this letter, we provide a point-by-point response to the Reviewers' comments (highlighted in blue), indicating the specific changes made. For convenience, we have included the new images and references directly within the response to facilitate the review process.

This revision was prepared in consultation with all co-authors, each of whom has approved the final version. We thank the Editor and the Reviewers once again for their valuable feedback.

Yours sincerely,
Ricardo M. Rosales, on behalf of all authors

Revision of manuscript PCOMPBIOL-D-25-02377

Journal Requirements:

If the reviewer comments include a recommendation to cite specific previously published works, please review and evaluate these publications to determine whether they are relevant and should be cited. There is no requirement to cite these works unless the editor has indicated otherwise.

We have carefully evaluated the suggested studies and, where relevant to the manuscript's technical scope and context, we have incorporated the citations.

1) We ask that a manuscript source file is provided at Revision. Please upload your manuscript file as a .doc, .docx, .rtf or .tex. If you are providing a .tex file, please upload it under the item type u2018LaTeX Source Fileu2019 and leave your .pdf version as the item type u2018Manuscriptu2019.

The documents have been provided as indicated.

2) Please upload all main figures as separate Figure files in .tif or .eps format. For more information about how to convert and format your figure files please see our guidelines:

We converted all Figures to .tiff format.

3) Some material included in your submission may be copyrighted. According to PLOSu2019s copyright policy, authors who use figures or other material (e.g., graphics, clipart, maps) from another author or copyright holder must demonstrate or obtain permission to publish this material under the Creative Commons Attribution 4.0 International (CC BY 4.0) License used by PLOS journals. Please closely review the details of PLOSu2019s copyright requirements here: PLOS Licenses and Copyright. If you need to request permissions from a copyright holder, you may use PLOS's Copyright Content Permission form. Please respond directly to this email and provide any known details concerning your material's license terms and permissions required for reuse, even if you have not yet obtained copyright permissions or are unsure of your material's copyright compatibility. Once you have responded and addressed all other outstanding technical requirements, you may resubmit your manuscript within Editorial Manager. Potential Copyright Issues: i) Please confirm (a) that you are the photographer of 1D, or (b) provide written permission from the photographer to publish the photo(s) under our CC BY 4.0 license.

We have attached PLOS's Copyright Content Permission form signed by the copyright holder of the photographs in Figure 1D. Moreover, Figure 1 was updated. Now it contains a combination of original hand-drawn elements, our own images, and open-source icons. We have updated the figure to ensure all external elements are from public domain sources. Below are the specific sources and licensing details:

- Emitting light: Replaced with `https://openclipart.org/detail/185271/light-bulb-icon-2`.
- Camera: Replaced with `https://openclipart.org/detail/343549/camera`

4) Please send a completed 'Competing Interests' statement, including any COIs declared by your co-authors. If you have no competing interests to declare, please state "The authors have declared that no competing interests exist". Otherwise please declare all competing interests beginning with the statement "I have read the journal's policy and the authors of this manuscript have the following competing interests"

We have added a completed "Declaration of competing interest".

Response to Reviewer's comments

Reviewer 1:

This study describes an expansive study of VT susceptibility in the context of EHT implantation using computational models of porcine hearts with MI induced by either LAD or LCx occlusion. The findings indicate that intrinsic maturation of the EHT patch (as represented by increased internal conductivity) and EHT patch location with respect to the scar/BZ are key factors in modulation of VT inducibility. In general, the authors' vivid descriptions of side-by-side simulations (e.g., with or without EHT, or comparing the two EHT locations) provide rich mechanistic insights on factors mitigating or exacerbating the inducibility of reentrant VT in these models. Independent of the main investigational thrust of the paper related to EHTs, the results presented reaffirm the fundamental importance of incorporating a plausible representation of the CS in bi-ventricular models in order to accurately represent the inducibility of reentrant arrhythmia (or the lack thereof) in the context the post-MI landscape. The authors have gone to great pains to exceed the standard of the cardiac EP modeling field in terms of experimental reproducibility by providing links to all relevant source code and repositories containing complete computational models that could plausibly be downloaded and re-used by other research teams with an appropriate amount of effort.

In general, the manuscript is well written and the computational work is exceptionally rigorous. The findings are noteworthy and will move the field forward. The presentation of simulation data alongside experimental findings is exciting. As enumerated below, there are a few major areas (and several other minor points) where I believe the manuscript could be improved, but these are generally intended as constructive feedback and should not be interpreted as a lack of enthusiasm on my part.

We are grateful to the Reviewer for the revision and for the helpful and constructive comments, which we addressed as follows.

Major Feedback:

1) In much of the manuscript, the matching of experimentally observed and simulated reentrant VTs is overstated. The second paragraph of the Discussion section is appropriately calibrated because it highlights the fact that the matching is qualitative in nature, and stresses that there are no experimental data presented corresponding to the critical simulations (i.e., those including EHT patches). Also, the validation study was performed on the simulations conducted in models without the CS, which is surprising given the major importance ascribed to the CS's presence elsewhere in the study. Finally, there were relatively few incidences of sVT in the simulations. Most of the episodes of reentrant arrhythmia observed were nsVT. The authors do not state until late in the Discussion that the majority of arrhythmias observed in optically-mapped hearts were sVT. Despite these issues, the qualitative validation itself is undoubtedly impressive and should be foregrounded accordingly. However, ensure that the findings are properly appreciated by readers, the authors are encouraged to be more explicit about the nature of the validation data (qualitative, limited to simulations of VT in MI models without the CS, differences in sVT/nsVT, etc.) and this information should be reported throughout the paper (esp. in Results).

We agree with the Reviewer that the details of experimental validation should be clarified early on. In order to provide precise context and properly describe the level of the validation, we have introduced the concept of the matching being qualitative in nature throughout the revised manuscript:

- Abstract: "*In silico* reentrant activity qualitatively reproduced the arrhythmic patterns observed experimentally in porcine MI models."
- Author summary: "We first achieved good qualitative agreement between our *in silico* simulated and the experimentally observed arrhythmic patterns in pigs."
- Introduction: "First, we qualitatively compared simulated arrhythmic dynamics with experimentally observed reentrant patterns obtained through optical mapping (OM)."

- Section 2.5: "To qualitatively validate our models, the simulated depolarization patterns calculated for LAD pig models were compared with those obtained from OM experiments for the same pigs when the experimental AIP described in Section 2.2.1 was applied."
- Section 3.1: "The experimental arrhythmic patterns mapped by OM in LAD pigs were qualitatively compared with the results from G1 simulations using the CS-BiV models of pigs 8-12" and "In spite of minor differences, experimental and simulated reentrant dynamics showed good qualitative agreement in all cases."
- Section 4.1: "*In silico* models qualitatively reproduce experimental porcine-specific arrhythmic dynamics" and "Our *in silico* simulations qualitatively reproduced the experimental dynamics observed in OM recordings of Langendorff-perfused LAD porcine hearts, which consistently exhibited VT induction, predominantly sVT."
- Conclusion: "Simulated arrhythmic patterns showed good qualitative agreement with the experimental data."

Moreover, as acknowledged by the Reviewer, the qualitative validation study (G1 simulations) was indeed performed with the CS incorporated into the simulations. To clarify this for the reader, we have updated the following:

- Section 2.5: "In the first group of simulations (G1), the complete AIP was applied to all CS-BiV models."
- Section 3.1: "The experimental arrhythmic patterns mapped by OM in LAD pigs were qualitatively compared with the results from G1 simulations using the CS-BiV models of pigs 8-12. Results for pigs 8, 10, and 11 are shown in Fig 4, where the presence of the CS is visible upon magnifying the simulation images."

Finally, we agree that the discrepancy between experimental and simulated VT sustainability should be explicitly stated in the Results. We have modified Section 3.1 to read: "While experimental VTs were sustained in all infarcted pigs, the corresponding *in silico* simulations exhibited a mix of dynamics: nsVTs were observed in models for pigs 8-10, whereas sVTs were successfully reproduced in pigs 11-12".

We have also extended the discussion regarding these sustainability discrepancies and the slower computational reentrant activity in Section 4.1: "In simulations, nsVTs were observed in pigs 8-10, whereas sVTs were reproduced in pigs 11-12, consistent with experimental observations in which sVT was always induced. We attribute the absence of sustained VT in pig models 8-10 to the more intensive experimental induction protocol, which used a greater number of pulses and a wider range of consecutive S2 trains than the simulations, although it was delivered at fewer anatomical locations"; and "The reduced reentry speed observed in simulations compared with experimental rotors (Fig 5 and S1 Video) likely reflects the use of averaged conductivity across the HZ and BZ and across models. This spatial homogenization may have reduced local CVs relative to the heterogeneous structure of the experimental tissue. To define BZ conductivity, which was generalized to all LAD and LCx models, we averaged the individual conductivity values required to computationally reproduce the CV observed in the MI region of the five LAD pigs, as previously characterized by OM under sinus and paced rhythm [1]. Subject-specific parameterization was not feasible for LCx pigs due to a lack of experimental data [1]. Similarly, CS distributions were assigned based on spatial benchmarks but lacked subject-specific experimental validation [1]."

We concluded this section with an encompassing summary: "Despite these limitations, the G1 simulations qualitatively captured the experimental arrhythmic dynamics without individualized adjustment of electrophysiological parameters or induction protocols, supporting the robustness of the modeling framework. Future studies will assess whether replicating the exact experimental pacing sequence *in silico* and performing fine-grained, subject-specific electrophysiological calibration (e.g., using OM under VT) enhance the stability of reentrant circuits in models 8-10 and better reproduce the reentry speed observed in all LAD models."

By implementing these changes, we believe the context, limitations, and level of validation are now clearly identified for the reader from the early stages of the manuscript.

2) Some rationale should be provided on why the specific S2 values were chosen. In the context of the original paper that motivated this protocol (Arevalo et al. 2016), multiple extrastimuli were given (S2, S3, S4) and the coupling interval preceding each extrastimulus was systematically reduced until the shortest possible while maintining capture. This protocol is not too burdensome to implement computationally since it can be easily achieved by creating a large number of checkpoints after each preceding stimulus, then running a very short stimulus for each possible extrastimulus to assess whether capture occurs. At minimum, this should be discussed as a potential limitation, since in some situations the window of vulnerability for VT initiation might be very narrow (e.g., in some cases presented here, an S2 of 270 or 275 ms might induce reentry in a model otherwise reported as completely non-inducible).

We thank the Reviewer for this constructive comment. We agree that the selection of specific S2 values and the implementation of a streamlined pacing protocol require clear justification and discussion. To address this, we have expanded Section 4.2 to explicitly contrast our protocol with the one established by Arevalo et al. [2]. This section now details the physiological and computational rationale behind our modifications, supported by contemporary evidence from the literature. The revised opening of Section 4.2 reads as follows:

"Arrhythmia inducibility was assessed using an *in silico* AIP inspired by previous approaches, such as the Virtual Heart Arrhythmia Risk Predictor [2], but streamlined by employing fewer pacing sites, a shorter S1 train, and a single S2 stimulus delivered at four coupling intervals to balance physiological fidelity and computational tractability. Specifically, Arevalo et al. [2] applied eight S1 stimuli followed by three trains (S2-S3-S4) of premature extra-stimuli. In their study, each premature train was initially delivered 300 ms after the last S1 stimulus and shortened in 10 ms steps until loss of capture or arrhythmia induction. In contrast, we used four S1 stimuli followed by a single premature S2 stimulus (at 250, 265, 280, or 295 ms). The motivation for this reduction was that, under deterministic modeling, electrophysiological activity stabilizes after only a few beats, thereby justifying a shorter pacing sequence. In line with this, Azzolin et al. [3] showed that as few as two stimuli can induce arrhythmia when delivered at the effective refractory period of the pacing site, and that additional premature stimuli rarely increased arrhythmia inducibility in computational atrial models. Accordingly, our protocol prioritizes identification of inducible substrates over exhaustive characterization of the full vulnerability window.

Furthermore, we adopted a more targeted distribution of pacing sites compared with the 19 locations (RV apex, RV outflow tract, and 17 AHA-based sites) evaluated by Arevalo et al. [2]. Our protocol used a subset of at least 5 pacing sites selected from those 19 locations. Because the BZ constitutes the critical substrate for reentry [4, 5, 6], pacing sites were distributed homogeneously around the MI border, as more distant pacing often elicits redundant reentrant patterns. Consistent with this, a comprehensive AIP applied to the atria showed that electrophysiologically remodeled regions exhibit greater arrhythmia susceptibility and that a reduced number of homogeneously distributed pacing sites can capture up to 90% of induced arrhythmias compared with more exhaustive protocols [3]. Overall, these strategic simplifications enabled high-throughput assessment of the CS-BiV complex while preserving substantial computational scale, yielding reentrant patterns that closely resemble experimental observations (see Section 3.1).

The selection of S2 coupling intervals was informed by both established literature [2, 7, 6, 8] and the specific electrophysiological constraints of our porcine BiV models. While Arevalo et al. [2] utilized a sequence of premature stimuli, initially delivered 300 ms after the last S1 stimulus with 10 ms decrements, other studies adapting this method to specific porcine or human phenotypes have employed S2 values starting at 250 ms [7, 6, 8]. In our study, due to HZ/BZ refractoriness, as defined by the Gaur et al. [9] cellular model and its experimentally calibrated tissue properties [1], short S2 intervals or near-MI pacing often failed to capture, preventing VT induction. Longer S2 delays increased inducibility, and variability in such inducibility across S2 values highlighted the value of selecting the shortest effective S2 and identifying the S2 delay that produces the highest inducibility for post-MI remuscularization analyses (G3 and G4 simulations). This establishes a robust framework for assessing inducibility with the Gaur et al. [9] model embedded in BiV simulations."

Furthermore, we have added the following text to the Section "Limitations, uncertainties, and future work":

"***In silico* AIP:** The combination of a limited, low-resolution S2 range and a reduced number of pacing sites, rather than a high-resolution exploration of coupling intervals or a complete 19-site sweep, may fail to capture narrow spatiotemporal windows of vulnerability for VT induction. Consequently, models classified as non-inducible under our protocol could potentially exhibit reentry under a more exhaustive coupling-interval search. Thus, although our *in silico* AIP qualitatively reproduced subject-specific experimental VT patterns with substantially lower computational cost, future studies may benefit from more spatially detailed and temporally extended stimulation strategies. From a spatial standpoint, although no fully automated quantitative criteria were used for pacing-site selection, we implemented a reproducible strategy designed to homogeneously encircle the MI region and assess proarrhythmic BZ heterogeneity [5, 4, 3]. Future work could incorporate automated methods based on geodesic distance to scar tissue or the identification of slow-conducting isthmuses, as proposed by Campos et al. [8]. Temporally, broader and site-specific S2 coupling interval ranges could be examined using checkpointing strategies and automated estimation of the effective refractory period at each pacing site, following approaches similar to those described by Azzolin et al. [3]. Collectively, these methodological refinements would further standardize the AIP and enable systematic identification of the key spatial and temporal pacing features underlying arrhythmia induction, while maintaining computationally feasibility. Such advances may uncover reentry mechanisms that arise only within narrow temporal windows or specific trigger-substrate configurations."

3) The discussion of post-implantation electrophysiological integration of EHTs and ventricular myocardium (esp. regarding ref. 60, doi: 10.1113/JP284244) is somewhat incomplete. A fundamental limitation of continuum models of cardiac EP is that it is challenging to represent heterogeneous coupling at the boundaries between two regions with distinct electrical properties. It would be helpful if the authors could speculate on how heterogeneity in electrical coupling between the patch and the underlying myocardium might affect the dynamics of reentrant VT inducibility, especially given observations from other types of post-MI cell therapy (e.g., predominantly focal VT onset a few days post-injection, then relief from VT risk several weeks after treatment, as reported by Liu et al. doi: 10.1038/nbt.4162)

We thank the Reviewer for highlighting these critical aspects of EHT-host integration. We agree that the continuum representation of the EHT-myocardium interface is an approximation of a more complex, heterogeneous biological boundary.

As noted in our "Limitations, uncertainties, and future work" section, we have already discussed, in our "Extrinsic factors post-implantation of the EHT" part, how incomplete physical engraftment and heterogeneous attachment (generated via air bubbles, stitching, immune EHT isolation, and shear stress) can influence VT risk, citing recent computational work [10, 11, 12]. However, to address the Reviewer's point regarding the limitations of continuum EHT-myocardium coupling and the specific dynamics reported by Liu et al. [13] in the context of the reentrant mechanism, we have expanded our "Degree of attachment" and "Transitional phase" points to include these physiological observations and the challenge of representing heterogeneous boundary coupling. The revised part is:

"**Extrinsic factors post-implantation of the EHT:** External influences also affect remuscularization outcomes [14, 15, 16, 13, 10].

- Degree of attachment: Incomplete physical engraftment may result from air bubbles in poorly adhered constructs, scar tissue from stitching, immune-mediated EHT isolation in xenotransplantation, or shear stress during cardiac contraction-relaxation cycles [10, 15]. While our modeling of BiV-EHT coupling as a continuum interface provides a tractable framework for organ-scale VT assessment, representing the discrete, heterogeneous electrical coupling at the graft-host interface remains a key challenge. As noted by Gibbs et al. [17], spatiotemporal heterogeneities in interface coupling can create localized source-sink mismatches that strongly influence focal arrhythmia susceptibility. Prior simulations suggest that homogeneous partial engraftment increases VT risk, whereas heterogeneous partial versus complete engraftment shows comparable risk [10, 11]. Consistently, our previous computational study found only minor electrophysiological variations across degrees of heterogeneous engraftment, provided minimal electrical contact existed [12]. Therefore, we focused on complete engraftment in this work.
- EHT-induced ectopy: Experimental studies report a high ectopic burden following cellular injection [16, 18, 13, 10]. Computational evidence indicates that ectopy arises under incomplete

engraftment or extreme hiPSC-CM heterogeneity, but not under complete engraftment. Sustained VT further requires a bradycardic beat (or absence of other non-EHT pacing) [19, 17] and suppression of CS retrograde conduction [19]. Our results and others [10, 11, 20, 12, 21, 22] found no ectopic-driven VT, consistent with the high electrotonic load of the host myocardium suppressing spontaneous activity and the intrinsically slower EHT beating rate, ensuring pacing by the host myocardium. This effect persisted despite using the most recent Paci et al. [23] model, which features enhanced calcium handling and more robust spontaneous beating. Rapid host-induced pacing may further suppress hiPSC-CM automaticity by depleting sarcoplasmic reticulum calcium stores, aligning with findings by Riebel et al. [19]. Recent preclinical and clinical EHT studies [18, 24] reporting no arrhythmogenicity support these observations. However, confirmation of stable EHT-myocardium coupling and high cell survival remains necessary. Future studies could investigate bradycardic rhythms and heterogeneous grafts to model conditions more favorable to ectopy.

- Transitional phase: We, like others [10, 11], focused on baseline MI and post-EHT implantation endpoints. In this context, our previous work [22, 12, 21] showed that the host myocardium dominates both depolarization and repolarization of the EHT once minimal engraftment occurs. Specifically, APD values within the EHT mirrored adjacent myocardial regions. Intrinsic automaticity was suppressed even with the inward, hyperpolarization-activated funny current in the updated Paci et al. [23] model, and CV within the EHT followed that of neighboring myocardium, consistent with prior *in silico* reports [10, 11, 20]. Nevertheless, dynamic processes during graft integration, such as temporal changes in EHT-myocardium coupling and cell viability (not considered here), may substantially influence arrhythmogenicity. Liu et al. [13] reported early focal VT risk days post-injection, which transitions to relative stability over weeks. Similarly, computational studies show that focal arrhythmia susceptibility waxes and wanes as graft-host coupling gradually increases [17]. For reentrant mechanisms, spatiotemporal variations in electrical coupling may either facilitate or obstruct formation of low-conduction isthmuses (substrates for unidirectional block) and the sustainability of reentrant circuits, resulting in a highly variable VT burden during integration. Future simulations could incorporate time-varying conductivity, stochastic boundary coupling, and hiPSC-CM electrophysiology to capture this high-risk transitional phase between implantation and full electrical integration."

4) The authors have used a pig ionic model (Gaur et al.) to represent action potential properties in the normal and BZ mycardium, but the Purkinje model (Stewart et al.) was calibrated based on human EP and the EHT properties (Paci et al. 2020) are based on hiPSC-CM. These limitations are justifiable for several reasons (e.g., there is no pig Purkinje model; implantation of pig-based PSC-CM patches is clinically irrelevant for clinical reasons), but should be more clearly presented by the authors (e.g., it should be clearly stated in Methods that the Stewart model is human-based). It would also be helpful for the authors to comment on the potential implications of these inter-species differences in the model components in terms of how their results should be interpreted.

We thank the Reviewer for this important suggestion. We agree that the provenance of modeling data should be explicitly stated and that the use of cross-species parameters warrants a detailed discussion regarding their impact on results interpretation.

While our BiV models integrate multi-modal porcine experimental data as described in our previous work [1], certain parameters were indeed adapted from human or canine literature where porcine-specific data are currently unavailable. In the revised manuscript, we have clarified these instances and expanded our discussion on the implications of these cross-species differences:

- Action potential spatial heterogeneities (Section 2.1): We now state that the transmural proportions (endocardium, midmyocardium, and epicardium) of 40:35:25 follow established human-related literature [25, 26]: "Transmural heterogeneities were implemented by solving the steady-state diffusion (Laplace) equation with varying boundary conditions [27]. In accordance with established human studies [25, 26], the endocardium, midmyocardium, and epicardium were defined using a 40:35:25 proportion."

- CS Electrophysiology (Section 2.4): We have explicitly noted that the Stewart et al. [28] model is human-based: “The biophysically detailed cellular models of Gaur et al. (pig) [9] and Stewart et al. (human) [28] were used to represent the AP of the porcine BiV models and their corresponding CS [1].”

- Myocardium and CS Conductivity (Section 2.4): We clarify that while the longitudinal diffusion coefficients (LDCs) for the healthy zone and CS were initially defined from human data [29, 30], they were successfully validated by replicating porcine-specific OM-derived CVs and activation times: “The HZ LDC was initially defined based on human studies [29]. However, this value was subsequently calibrated and validated using OM-derived conduction velocities (CVs) from the anterior surface of the porcine heart [1]. Similarly, although the CS LDC was set to achieve CVs characteristic of the human CS ($\approx$ 200 cm/s) [30], it successfully reproduced the experimental, OM-derived maximum activation times and depolarization patterns of the anterior porcine heart under sinus rhythm [1]. The LDC for the CS was reduced near its endpoints using a sigmoid profile to match the ventricular LDC, as proposed by Dux-Santoy et al. [31], thereby enabling retrograde propagation at the PMJs. Bidirectional conduction across the PMJs was achieved (see S1 Figure). A minimal anterograde propagation delay of 1.1 ms was observed, whereas the retrograde delay was zero (see discussion in S1 Section and Section 4.6).”

- BZ Excitability (Section 2.4): We specify that the reduction of the fast Na^+ current follows canine-related experimental observations of post-MI remodeling: “The excitability in BZ was reduced by decreasing the conductance of the fast Na^+ current to 38% of its default value. This adjustment follows canine-related experimental observations [32] and aligns with established protocols from previous human and porcine computational studies [2, 33, 6].”

To justify the use of these parameters and discuss their potential implications on VT inducibility, we reference the sensitivity analysis conducted in our previous study [1]. This analysis demonstrated the following:

- Robustness to action potential duration (APD) and conductivity variations: Variations of $\pm$10% in APD heterogeneity ratios and $\pm$20% in myocardial/CS conductivities had negligible impacts on global electrocardiographic morphology and tissue-level metrics (activation times and conduction velocities). This confirms that the model is not critically sensitive to moderate deviations in these human-derived parameters.

- Sensitivity to I_{Na} remodeling: Conversely, the model showed higher sensitivity to BZ G_{Na} variations ($\pm$6%), which induced up to a 10% change in CV within the BZ.

To reflect these findings and their implications for VT inducibility, we have added a comprehensive discussion to the “Limitations, uncertainties, and future work” (Section 4.6):

“Several parameters were derived from human or canine studies that reproduce the qualitative electrophysiological behaviors typical of large mammalian hearts [25, 26, 28, 30, 34, 32, 29, 2, 33]. In our previous work [1], we performed a sensitivity analysis of regional APD ratios, myocardial and CS conductivities, and post-MI I_{Na} remodeling to evaluate the impact of these non-porcine parameters on global electrophysiological characteristics. Variations in APD heterogeneities and conductivities had minimal effects at the tissue and organ levels, whereas small changes in I_{Na} produced substantial alterations in CV within the BZ. In this context, Campos et al. [35] identified CV slowing as a key determinant of reentrant VT probability. These findings highlight the importance of calibrating sodium current remodeling using porcine-specific post-MI data to reduce model uncertainty. Overall, our results indicate that most cross-species parameter choices preserve model robustness, although certain variables may introduce localized bias. While current parameters were calibrated against porcine-specific OM and electrocardiogram data to ensure physiological consistency, future availability of more detailed experimental datasets would enable further refinement and reduce residual cross-species uncertainty.”

5) No specific rationale is presented for setting the EHT conductivity values to exactly 10% and 90% of the values used in the HZ for the immature and mature EHTs, respectively. The authors should articulate how they arrived at these values. Moreover, it should be emphasized that the functional

consequence of randomizing fiber orientation in the EHT regions is that the effective conductivity would be much lower than for organized, laminar myocardium even in the absence of conductivity modification.

We thank the Reviewer for the opportunity to clarify the rationale behind our EHT conductivity selections and the representation of EHT's cardiomyocyte orientation.

Rationale for EHT Conductivity Values: The selection of 10% and 90% conductivity values for immature and mature EHTs, respectively, was based on the following criteria:

- *Immature EHT (10%):* This value was selected to match conduction velocities observed in our previous experimental and computational studies of *in vitro* EHTs [22, 15, 14]. In Iglesias-García et al. [15], we demonstrated that an LDC of 0.00013 cm^2/ms (10% of the value set for healthy tissue) yielded a conduction velocity of ≈12 cm/s. This highly aligns with experimental OM measurements of EHTs, where velocities ranged between 5 and 24 cm/s depending on the scaffold architecture and the post-fabrication culturing time [15, 14]. In the manuscript, we added to Section 2.5: "In all G3 simulations, the conductivity for immature EHTs was set to 10% (C10) of the value found in the HZ (0.00013 cm^2/ms) to match experimental observations. This value was previously validated in studies where simulated maximum activation times and CVs (≈12 cm/s) closely matched OM measurements from *in vitro* EHTs [22, 15]. Experimentally measured velocities typically range from 5 to 24 cm/s depending on scaffold architecture and EHT culturing [15, 14]."
- *Mature EHT (90%):* The use of a high conductivity (90% of healthy tissue) represents a "best-case" maturation scenario. Previous computational studies have emphasized that while conductivity should not exceed that of the healthy myocardium, matching it as closely as possible is critical for reducing arrhythmogenic risk [20, 10, 11, 22, 12, 21]. Such higher conductivities are increasingly achievable through advanced scaffold designs (e.g., diamond-shaped pores) that improve cell alignment and maturation via directional stiffness control [15], or the use of electroconductive fabrication materials [36, 37]. In the revised Section 2.5 we have added: "Setting the S2 stimulus at 295 ms for this pacing site, all coupled BiV-EHT models, with EHT located at L1 and L2, were evaluated with the EHT conductivity set to 90% (C90) of the HZ value. This conductivity represents a mature, highly integrated scenario. Computational evidence indicates that matching the host myocardium's conductivity is critical to prevent the EHT from acting as a proarrhythmic substrate [20, 10, 11, 22, 12, 21]. Such high functional maturation is increasingly achievable through advanced scaffold designs, including diamond-shaped pores for directional stiffness control or the incorporation of electroconductive materials, which enhance cell alignment, connexin-43 expression, and electrical integration [15, 36, 37]. Consequently, results from C10 (immature, low conductivity) and C90 (mature, high conductivity) EHTs were compared for each subject to quantify the effect of post-implantation electrical maturation on overall VT risk."

EHT Fiber Orientation and Isotropy: We agree with the Reviewer that disorganized cell alignment significantly reduces effective conduction compared to laminar myocardium. To avoid confusion regarding our modeling approach, we have clarified that the EHT was modeled as an isotropic tissue (transversal-to-longitudinal ratio of 1.0). Since we did not implement a preferential conduction axis, we have removed references to "random fiber orientation" and updated the description in Section 2.4 to: "EHT conductivity was set as completely isotropic due to the lack of precise data on connexin-43 distribution in hiPSC-CM cultures; consequently, the EHT was modeled without preferential directionality or a defined fiber architecture". Additionally, in the "Limitations, uncertainties, and future work" section under "Cell alignment", we have modified the text to reflect this: "Here, a disorganized distribution was assumed for hiPSC-CMs in the EHT, consistent with prior slab and BiV simulations showing a limited arrhythmic impact of alignment [22, 21, 10]". Moreover, a new Section "Simulated conduction velocities" presents the *in silico* computed CVs for all conducting tissue regions and how these relate to experimental porcine-specific myocardial and *in vitro* EHT measurements. Specifically for the EHT, this Section reads:

"The CV in the EHT was influenced by both its intrinsic conductivity and the underlying myocardium, suggesting that the host tissue effectively drove EHT depolarization, as reported in [10, 12,

21]. At the L1 position with low conductivity (C10), the EHT exhibited a CV of 13.6 cm/s, consistent with reported *in vitro* EHT values (5–24 cm/s) [15, 14]. Because the EHT at this location predominantly overlies non-conducting SZ or slow-conducting BZ, the simulated CVs naturally approximated *in vitro* values. In contrast, when assigned adult-like conductivity (C90), the CV in the EHT increased, particularly at the L2 position where it overlies fast-conducting HZ, approaching healthy myocardial velocities.

Overall, this analysis confirms that the simulated CVs accurately reproduce porcine-specific experimental values under healthy and MI conditions, in both sinus and paced rhythms, as well as *in vitro* EHT dynamics, despite the non-uniform, bimodal mesh resolution. These results provide a solid foundation for our arrhythmia inducibility assessment.

Minor Points:

6) When the different rounds of simulations are presented in the Methods, it is initially unclear whether the G1 simulations are run with or without the CS. This becomes obvious when the Results are read, but it would be helpful to specify this more explicitly in Methods.

We agree with the Reviewer. As highlighted in the answer to the Reviewer's first comment, the qualitative validation study (G1 simulations) was indeed performed with the CS incorporated into the simulations. To clarify this for the reader, we have updated the following:

- Section 2.5: "In the first group of simulations (G1), the complete AIP was applied to all CS-BiV models."
- Section 3.1: "The experimental arrhythmic patterns mapped by OM in LAD pigs were qualitatively compared with the results from G1 simulations using the CS-BiV models of pigs 8-12. Results for pigs 8, 10, and 11 are shown in Fig 5, where the presence of the CS is visible upon magnifying the simulation images."

7) "Thus, this constitutes one of the largest and most complex cardiac electrophysiological simulation studies to date". I'm not sure it's valuable to use superlatives like this. It is certainly a large study of computational cardiac EP compared to some others. Quantitatively gauging the extent to which it is more or less "complex" than others is another matter entirely. I would instead suggest the authors revise this phrasing to correctly emphasize that the computational burden (in terms of compute wall-time required) and complexity of meshes (in terms of finite element average edge length, incorporation of EP heterogeneity) are cutting edge with regards to other comparable studies (e.g., recent organ-scale EP papers focused on hiPSC-CM integration by Yu et al. [2019 Sci Rep, 2022 Cardiovasc Res] and Riebel et al. [2025 preprint, doi: 10.1101/2025.10.03.680241])

We thank the reviewer for this constructive suggestion. We agree that using superlatives such as "largest and most complex" can be subjective. We have revised this phrasing to more precisely highlight the computational scale and the technical advancements of our study. Specifically, we now emphasize that the computational burden, the mesh resolution, and the incorporation of multi-scale electrophysiological heterogeneities align with current cutting-edge research in the field, such as the work by Yu et al. [10] and Riebel et al. [19]. Furthermore, we have clarified that our modeling of the EHT-myocardium interface represents a significant advancement in terms of anatomical and physiological realism for organ-scale simulations.

The revised ending of Section 2.5 is: "In total, 335 simulations were performed, representing a cumulative duration of more than one million milliseconds of electrophysiological activity. Although large-scale simulated time is increasingly common in HPC-based cardiac research, the scale of this study is particularly notable given the high spatial resolution of the BiV meshes and the dense incorporation of electrophysiological heterogeneities. The substantial wall-clock time required and the fine spatial discretization place this work at the forefront of current simulation studies on remuscularization [10, 11, 19]. Furthermore, by modeling the EHT as directly implanted onto the epicardial surface of the BiV anatomy, we introduce an enhanced level of structural and physiological realism. This detailed representation of the graft-host interface at the organ scale captures a level of complexity not previously addressed in simulations of EHT-mediated remuscularization [10, 11], thereby providing a

more robust framework for evaluating post-implantation arrhythmogenicity."

8) There are some consistency issues in S1_Video.mp4, in terms of apparent jumps in timing of frame assembly. For instance, in the case shown in the bottom-right corner (Pig 12), there is a discontinuity between the 12- and 13-second mark of the video where the spatiotemporal evolution of Vm(t) changes abruptly in a way that reflects non-physiological behavior. This is likely a issue of how the video was assembled. In general, all videos should be double-checked.

We thank the Reviewer for the observation regarding the video assembly. To improve the clarity and consistency of the supporting material, we have revised all four videos. In the original version of S1_Video, embedded videos of varying lengths were looped, which may have caused confusion. In the revised version, we have modified the sequences so that each embedded video displays its final frame once the simulation concludes, rather than restarting. Furthermore, we have revised all supporting videos by ensuring a seamless assembly and incorporating dashed lines to clearly show the EHT boundaries.

9) The summary data in Fig 5 (left panel) could be improved by adding information about NC sites (i.e., no-capture), which are also relevant in this context. The summary data in Fig 5 (right panel) should be disaggregated so that the individual IS values for each pig model can be appreciated by the reader. As presented, the inter-model heterogeneity is lost. Violin plots or box-and-whisker plots with all individual data points shown could work.

We agree with the Reviewer that Fig 5 (now Fig 6) could be improved for better clarity. Following the Reviewer's suggestion, we have incorporated the non-capturing pacing sites into the left panel of the figure. Furthermore, we have added a third panel on the right illustrating the subject-specific variation in VT inducibility across 265, 280 and 295 ms S2 intervals, providing a clearer representation of inter-model VT burden variability. The revised figure and its updated caption are provided below as Fig 1.

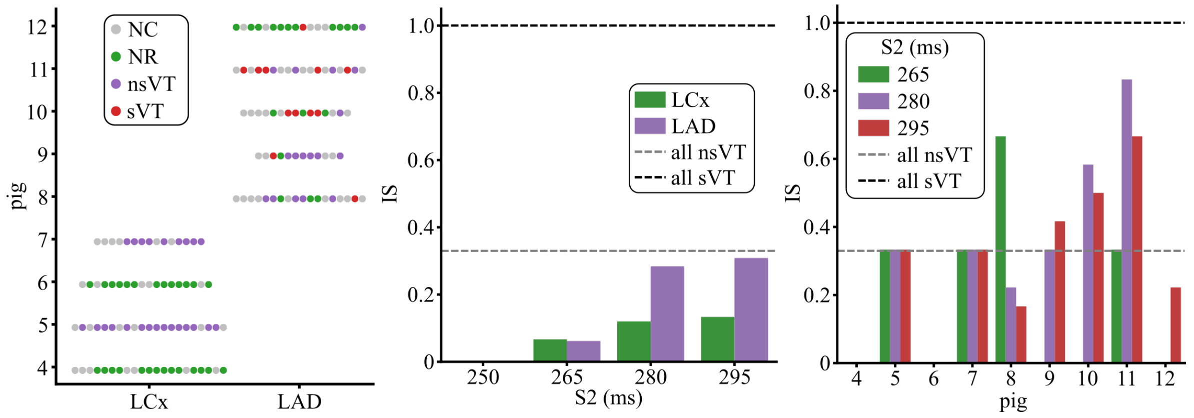

Figure 1: **Post-MI inducibility for all nine CS-BiV models when all pacing sites were tested (G1 simulations).** The left panel shows the pig-wise VT inducibility results as a function of MI location when S2 values of 265, 280, and 295 ms were used. The middle panel depicts the IS for both MI types as a function of all S2 values. The right panel presents the pig-wise IS for all S2 values. As detailed in Section 2.2.2, the simulations where the stimulation did not capture (NC) were not considered into the IS computation.

Additionally, we have updated the text in Section 3.2 to reflect these changes, including more frequent references to the new figure and noting the specific case of pig 8 (the only subject where VT burden decreased as the S2 interval increased from 265 to 280 ms): "Only in pig 8 was inducibility reduced as the S2 delay increased from 265 to 280 ms (Fig 6, right panel).

10) The "LI" vs. "HI" subsets of models tested in G2 vs. G1 were somewhat complex for me to grasp. It might be helpful to slightly tweak the way this is expalined in the Methods, and perhaps

highlight the corresponding columns in Supplemental Tables 1 and 2 where the identical results are presented. (i.e., for Pig 6, the "with CS" column in Table S2 is identical to the 295 column in Table S1).

Following the Reviewer's comment we enhanced the explanation of high- and low-inducible pig models in Section 2.5. The revised paragraph for describing G2 simulations is:

"In a second group of simulations (G2), proarrhythmicity was evaluated as a function of the inclusion or absence of CS in the models. Specifically, the LAD and LCx pigs with the highest (HI) and lowest (LI) VT burdens in G1 simulations were resimulated without the CS at an S2 interval of 295 ms. These results were subsequently compared to the reentrant activity observed in the corresponding G1 simulations, which included the CS, at the same S2 interval."

In accordance with the Reviewer's suggestion, we have extended the captions for Supporting Tables S2, S3, and S4 to explicitly identify which data are identical to results already presented in previous tables. The updated captions for these Tables are:

- **S2 Table. Results of the arrhythmia inducibility protocol obtained in the G2 simulations.** G2 simulations correspond to the assessment of the CS presence. LCx pigs (6-7) are shown at the top of the table, and LAD pigs (11-12) are shown at the bottom of the table. PS: pacing site, NC: no capture, NR: no reentry, nsVT: non-sustained VT, sVT: sustained VT. Note that results in column "with CS" are identical to the outcomes of G1 simulations (S1 Table) where an S2 interval of 295 ms was employed. Additionally, pacing sites which resulted in NC in the G1 simulations were excluded (-).
- **S3 Table. Results of the arrhythmia inducibility protocol obtained in the G3 simulations.** G3 simulations correspond to the assessment of the remuscularization and the location of the EHT implantation. LCx pigs (4-7) are shown at the left table, and LAD pigs (8-12) are shown at the right table. PS: pacing site, MI: baseline pre-EHT, L1: EHT at L1, L2: EHT at L2, NC: no capture, NR: no reentry, nsVT: non-sustained VT, sVT: sustained VT. Note that results in column "MI" are identical to the outcomes of G1 simulations (S1 Table) where an S2 interval of 295 ms was employed. Additionally, a low EHT conductivity was used, and pacing sites that resulted in NC in the G1 simulations were excluded (-).
- **S4 Table. Results of the arrhythmia inducibility protocol obtained in the G4 simulations.** G4 simulations correspond to the assessment of the EHT conductivity variation when the EHT was implanted in different locations. The left table correspond to the EHT location L1 and the right table correspond to the EHT location L2. LCx pigs (4-7) are shown at the top of the tables, and LAD pigs (8-12) are shown at the bottom of the tables. PS: pacing site, NR: no reentry, nsVT: non-sustained VT, sVT: sustained VT, C10/90: EHT with a conductivity equal to the 10/90% of the value found in the healthy adult-like tissue. Note that results in column "C10" are identical to the outcomes of G3 simulations (S3 Table) where an S2 interval of 295 ms and low EHT conductivity were employed.

11) In videos showing spatiotemporal evolution of Vm(t) in which EHTs are present, it would be very helpful to superimpose a dotted black link delineating the edges of the hPSC-CM region (as in Figs 9-10)

We thank the Reviewer for this helpful suggestion. As noted in our previous response, all videos depicting the EHT models (S3 Video and S4 Video) have been updated to clearly delineate the EHT boundaries with a dashed black line.

12) The use of the IS convention in the context of Fig 10 is somewhat confusing. Because the analysis is limited to pacing sites in each model that were guaranteed to induce at least nsVT for either L1 or L2 of the EHT, the metric has a different significance than in prior analyses (where inducibility was assessed across multiple pacing sites, many of which did not induce VT). The authors are encouraged to reconsider the use fo the metric in this context, since it artificially inflates the apparent arrhythmogenicity (i.e., values of 1.0 and 0.33 are common here, whereas they are rare in earlier analyses that aggregate data from multiple pacing sites).

We agree with the Reviewer that the use of IS in the G4 simulations was potentially misleading, as the single simulation per case made the interpretation of an averaging-like metric unclear. To address this, we have revised panels (c) and (d) in Fig 10 (now Fig 11). In the updated manuscript, the results of G4 simulations are now represented using a bar plot that categorizes outcomes into three discrete levels: NR, nsVT, and sVT. To maintain consistency with this categorical approach, we have replaced the term "IS" with "VT burden" in Section 3.5 when referring to these panels. The revised figure is provided below as Fig 2.

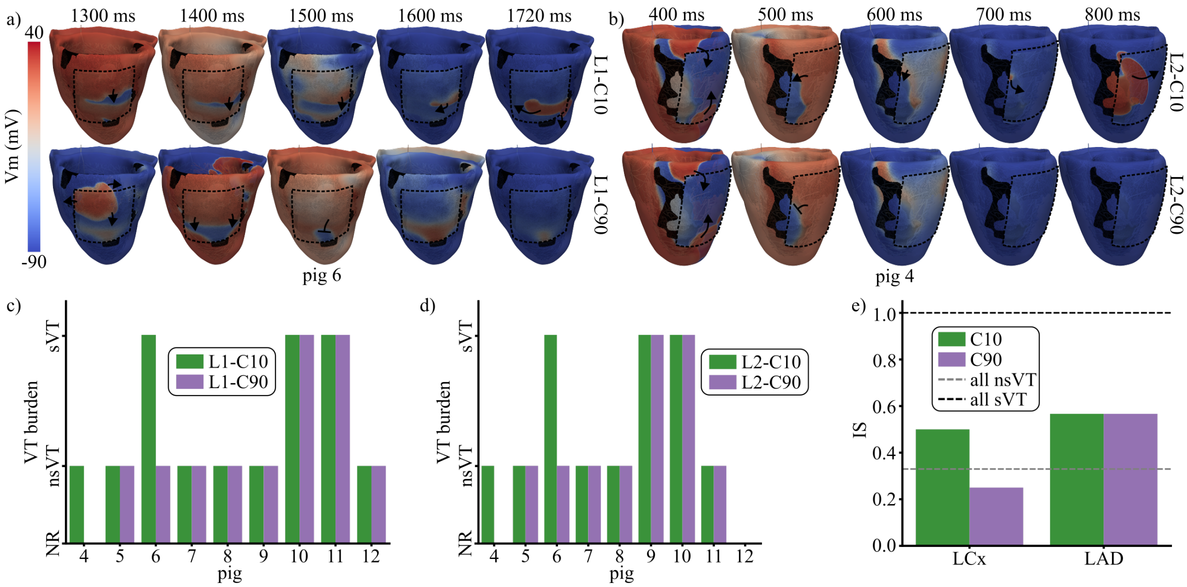

Figure 2: **Effect of EHT electrical maturation on arrhythmogenesis (G4 simulations).** AIP-derived electrical activity is shown for pig 6 (a) and pig 4 (b) when low (top) and high (bottom) EHT (dashed black line) conductivities were used. The pacing sites were 15 for pig 6 and 1 for pig 4. The timings refer to the time of application of the last S1 stimulus. The VT burden was obtained per pig using different EHT conductivities and locations L1 (c) and L2 (d) for a single pacing site. Panel e shows IS for conductivities C10 and C90 of the EHT in MI groups LCx and LAD.

13) The discussion of CS as a modulator of arrhythmia inducibility is excellent, but could be further supplemented with discussion of aspects of the VT substrate not included in the present model (e.g., transmural gradients in expression of channels like IKATP), which were examined in earlier generations of computational models incorporating the CS (see doi: 10.1016/j.hrthm.2013.08.010). If anything, these findings along with the new insights elucidated by the authors emphasize the criticality of including the CS in such models if they are to be used to predict clinical inducibility of VT.

In accordance with the Reviewer's suggestion, we have expanded our discussion in Section 4.3 regarding the mechanisms by which the CS modulates VT. We have specifically incorporated mechanistic insights providing a more comprehensive context for our findings. The following text has been added to the manuscript: "Boyle et al. [38] provided additional *in silico* mechanistic evidence of CS-mediated VT modulation. Specifically, they demonstrated that APD prolongation near the PMJs, a phenomenon consistent with our own observations [1], can induce varying local electrophysiological patterns, such as rotor anchoring and wavefront fractionation."

14) The authors should carefully consider whether the models can appropriately be described as "digital twins" (i.e., DTs). Use of this terminology is increasingly common, but in some cases it can be misleading and undermine the value of otherwise excellent computational modeling work. Bhagirath et al. (doi: 10.1093/europace/euae295) recently proposed a more nuanced nomenclature (e.g., functionally similar vs. functionally equivalent DTs). The present study lacks some important hallmarks of DTs (e.g., predicting emergent system behavior that the model was not specifically calibrated to reproduce). The absence of optical mapping data from hearts with implanted EHTs to corroborate

the simulation predictions also calls into question that aspect of the ”twinning”. To be clear, none of these issues are critical flaws, nor do they undermine the trustworthiness of the computational work; however, use of the DT terminology remains an issue.

We agree with the Reviewer that the term “digital twin” is inappropriate. To address this concern, we have replaced “digital twin” with “functionally similar digital twin”, as suggested by the Reviewer. In addition, we now refer to these models as “subject-specific *in silico* or computational models” rather than “digital twins”. We believe this terminology is more appropriate given the individualized nature of the BiV domain and myocardial infarction substrate in each pig model and the species-specific electrophysiological parameterization of all models, as described in detail in our previous work [1].

Reviewer 2:

Rosales et al. present an in silico study to assess arrhythmogenic risk after post-MI remuscularization with engineered heart tissue (EHT) patches. The authors integrate EHT geometries into nine validated vessel-specific porcine biventricular models (LCx, n=4; LAD, n=5) and evaluate VT vulnerability with an S1–S2 arrhythmia inducibility protocol across pacing sites, coupling intervals, infarct substrates, implantation locations, EHT conductivity (as a proxy for maturation), and the presence/absence of the ventricular conduction system (CS). The authors report (i) higher VT burden in LAD vs LCx infarcts, (ii) strong modulation of inducibility/dynamics by CS “shortcuts,” and (iii) an overall mitigating effect of higher EHT conductivity on VT burden, with implantation location effects depending on scar transmurality. I would like to highlight the well written manuscript and the attempt to connect model behavior to experimentally observed reentrant patterns (optical mapping) as strengths of the study. However, there are some concerns that should be addressed.

We are grateful to the Reviewer for the revision and for the helpful and constructive comments, which we addressed as follows.

Major comments:

Line 28, please add references to support the comment “A promising tissue engineering strategy under active investigation”.

In response to the Reviewer’s comment, we have added supporting references to this statement, which now reads as follows: “A promising tissue engineering strategy under active investigation is the remuscularization of the infarct scar using human-induced pluripotent stem cell-derived cardiomyocytes (hiPSC-CMs) to restore LV ejection fraction [18, 24, 39, 15, 40, 41, 14].”

Line 30, the author commented in plural that there are ”Recent studies” in plural but there is only one reference given in the manuscript. Please provide more than one reference to support this phrase.

In response to the Reviewer’s comment, we have added supporting references to this statement, which now reads as follows: “Recent studies have shown that the implantation of engineered heart tissues (EHTs) containing hiPSC-CMs can improve the post-MI ejection fraction in athymic rats with LAD-induced infarctions [15, 39].”

Line 46, while it is true that animal models are the foundation of basic research and different areas of investigation there are several studies in the field of cardiac modeling that use human data. Please add explicitly in the text why using human data could pose limitations and how animal experiments can overcome this limitation. Extend the phrase citing other studies that have been used to study ventricular arrhythmias and ischemia.

We thank the Reviewer for this insightful comment. We have revised the Introduction to explicitly acknowledge the limitations inherent in human-based cardiac modeling while clarifying the unique advantages offered by animal datasets. Specifically, we have expanded the discussion on the scarcity of high-resolution, multi-scale human data, where matched structural and functional datasets are rarely

available for the same individual; and on how animal models, particularly the porcine model, allow for the highly controlled, multi-scale validation required for complex therapy evaluation.

Furthermore, we have highlighted recent computational studies that utilize species-specific models to evaluate MI-related arrhythmogenicity and the mechanisms underlying ionic current blockade that lead to afterdepolarizations, while potentially diminishing *in vivo* preclinical experimentation. We also contextualize recent work on the computational assessment of post-MI EHT-mediated remuscularization, emphasizing that while human models are valuable for clinical translation, there remains a critical need for precise, species-specific preclinical computational studies to accurately interpret the large animal experiments that currently drive the field.

The revised text now reads:

"Over the past few decades, advances in biological data acquisition, computational power, and data storage have propelled the emergence of *in silico* modeling and simulation as a robust framework for investigating cardiac electrophysiology, particularly for therapy evaluation and diagnostic support [2, 10, 42]. Computational electrophysiology provides substantial advantages over traditional clinical studies, offering greater experimental flexibility while substantially reducing the time, cost, and resources required. While human-based models offer the highest clinical relevance, their development is often limited by the scarcity of high-resolution, multi-scale datasets obtained from the same individual. Acquiring matched anatomical and functional data in human cohorts is technically and ethically challenging, frequently necessitating the use of generic or population-based parameters [43, 1]. In contrast, animal models provide highly controlled experimental conditions that facilitate the acquisition of subject-specific data, enabling the development of precisely validated models essential for investigating complex phenomena such as cardiac resynchronization therapy, MI, and ventricular arrhythmogenesis [44, 45, 6, 1].

Domestic *Sus scrofa* pigs are considered the gold standard due to their close physiological similarity to humans, particularly in coronary anatomy, hemodynamics, and electromechanical properties [46]. Porcine computational models have been instrumental in characterizing the arrhythmogenic substrate following MI [6] and in elucidating mechanisms by which ionic current blockade produces early or delayed afterdepolarizations [9]. Regarding EHT-mediated epicardial remuscularization, recent studies by Yu et al. [10] and Fassina et al. [11] have employed human biventricular (BiV) and LV frameworks, respectively, to investigate the electrophysiological impact of this therapy. However, because most experimental feasibility studies for post-MI remuscularization are conducted in large animal models [16, 41, 18], there remains a critical need for precise, species-specific computational assessments to accurately interpret and translate these preclinical results."

Line 61, 73 and along the manuscript, the authors introduce the wording "comprehensive arrhythmia inducibility protocol (AIP). However, during the manuscript I did not understand how the used arrhythmia protocol is "comprehensive". Please provide sufficient evidence to support why the API is "comprehensive" otherwise I suggest removing it from the manuscript and just call it an "arrhythmia inducibility protocol".

We agree with the Reviewer that the term "comprehensive" may be an overstatement. Consequently, we have removed this descriptor from the revised manuscript and have consistently adopted the more precise term "arrhythmia inducibility protocol" throughout.

Line 89, please explain explicitly in the manuscript what is a multi-diffusion problem. Adding the explanation that different Laplace solutions are used to create gradients that are then used to create the different regions of the ventricular myocardium. Additionally, please add to the manuscript the values of the gradients used for each region.

We thank the Reviewer for this suggestion. We have revised the manuscript to explicitly define the multi-diffusion approach as the use of several independent solutions of the Laplace equation. Furthermore, we have included the specific proportions assigned to the endocardial, midmyocardial, and epicardial layers, as well as the apicobasal regions, ensuring the methodology is transparent and reproducible.

The revised text now reads: "Transmural heterogeneities were implemented by solving the steady-state diffusion (Laplace) equation with varying boundary conditions [27]. In accordance with estab-

lished human studies [25, 26], the endocardium, midmyocardium, and epicardium were defined using a 40:35:25 proportion. Additionally, an apicobasal gradient was defined by solving the Laplace equation with constant Dirichlet boundary conditions at the apex and base of the BiV domain. The BiV domain was subsequently partitioned into base, middle, and apex regions using an empirical proportion of 48:35:17 [1]."

Line 92-93, the conduction system is one of the main parts of the results and analysis of this manuscript. However, few details are given. For example, there are no descriptions about the number of PMJs connections to the myocardium, the resolution of the CS mesh, and the conduction velocity of the CS, etc. Additionally, there is no analysis of the CS construction and the impact on the depolarization, even if this is out of the scope of this study it should be mentioned in the discussion section and add future lines of work in this direction with the data available from the animal models.

We thank the Reviewer for highlighting the need for more granular detail regarding the CS. We acknowledge that while this study builds upon our previously validated CS architectures described in Rosales et al. [1], providing these specifics here is essential for the manuscript's completeness.

In the revised manuscript, we have included the requested details regarding PMJ density, CS mesh resolution, and conduction velocity. Furthermore, we have expanded the Discussion to address the impact of CS construction on depolarization and future directions for model refinement.

Specifically, we have implemented the following changes and additions:

- **PMJ connection and CS mesh resolution:** In Section 2.1, we expanded the CS description as follows: "The CS was generated for each model using a fractal tree algorithm and subsequently projected into the ventricular wall, following established methods [47, 48]. The CS mesh was defined using line elements with a mean length of 528 µm, incorporating an average of 1623 Purkinje-muscular junctions (PMJs). CS-activated ventricular nodes were located within a maximum distance of 0.5 mm from the CS endpoints, with an average of 14.5 connections between each endpoint and the myocardial nodes to avoid source-sink mismatch-mediated conduction blocks at PMJs [1]. Detailed descriptions of the BiV MI and CS model construction and their validation against porcine-specific experimental data are provided in our previous work [1]."
- **CV in the CS:** In Section 2.4, we included specific CV values and the methodology for CS-ventricular coupling: "Similarly, although the CS LDC was set to achieve CVs characteristic of the human CS ($\approx$ 200 cm/s) [30], it successfully reproduced the experimental, OM-derived maximum activation times and depolarization patterns of the anterior porcine heart under sinus rhythm [1]."
- **Bidirectional PMJ propagation:** In Section 2.4, we elaborated on the anterograde and retrograde delays observed at the PMJ. Specifically, we refined the description of this phenomenon within the text and included supporting S1 Section. This new section details our methodology for computing these delays and provides a comparative discussion of our results alongside previous simulation studies and experimental data. The revised text in Section 2.4 is as follows: "The LDC for the CS was reduced near its endpoints using a sigmoid profile to match the ventricular LDC, as proposed by Dux-Santoy et al. [31], thereby enabling retrograde propagation at the PMJs. Bidirectional conduction across the PMJs was achieved (see S1 Figure). A minimal anterograde propagation delay of 1.1 ms was observed, whereas the retrograde delay was zero (see discussion in S1 Section and Section 4.6).". This specific topic is presented in more detail in a following answer to the Reviewer.
- **CS construction analysis and future work:** In the "Limitations, uncertainties, and future work" section, we expanded the discussion to clarify that while the current architecture was validated against multi-scale experimental data (electrocardiogram and OM), future iterations will benefit from emerging porcine-specific data. The revised text now reads: "Accurate CS representation is confirmed to be essential for reducing variability in arrhythmia inducibility. However, precise individualization remains limited, as detailed CS anatomy can typically only be obtained from *ex vivo* histology, and a comprehensive porcine-specific electrophysiological cellular model is still lacking. Currently, endocardial mapping and non-invasive extracellular potential matching represent the most viable methods for validating CS models [33, 49, 50]. In Rosales et al. [1], we

validated the CS architecture used here against porcine subject-specific electrocardiogram and OM data, providing a thorough analysis of the CS architecture's effect on ventricular activation at tissue and organ levels under healthy and MI conditions. The established guidelines were followed for the individualized construction of the CS in all nine models used herein [1]. As additional data become available, porcine-specific functional (e.g., ionic dynamics, conductivity, and anterograde-retrograde PMJ delays) and structural (architecture, PMJ location) CS features should be integrated into the modeling framework to enable more robust preclinical modeling of CS influence on depolarization and arrhythmia. Furthermore, future work should incorporate post-MI region-specific CS conduction delays, recently shown to be highly arrhythmogenic by Sayers et al. [51]."

Line 98, please add a brief description of which dye was used and the concentration and which motion-blocker was used and at what concentration.

Following the Reviewer's comment, we have extended the description of the OM procedure. The revised text in Section 2.2.1 now reads: "Briefly, the heart of LAD pigs was excised and placed on a Langendorff apparatus for OM. After electromechanical uncoupling with 10 µM 2,3-butanedione monoxime in Tyrode's solution, a 100 µL bolus of the voltage-sensitive dye di-4-ANEPPS (excitation: 482 nm, emission: 686 nm; Biotium, Inc., Hayward, CA, USA) was infused for 5 min in 4.16 mM DMSO to monitor electrical activity via fluorescence changes. The spatial resolution was defined by a subject-specific conversion factor established before the OM experiments and maintained constant throughout the study (pigs 8–12: 0.081, 0.0729, 0.0860, 0.0885, and 0.0906 cm/pixel, respectively). All recordings were acquired at a temporal resolution of 2 ms."

Lines 116-119, please provide more details for reproducibility of the study on "by manually selecting at least 5 of the 19 pacing sites, 118 with the criterion that they were well distributed around the MI". The reasoning of reducing the computational time could be a valuable reason but this manual selection makes it hard to reproduce the AIP and might benefit future studies on the induction of arrhythmias. Could there be a more reproducible approach to select the 5 pacing sites, such as distance to the scar area which one can think of when looking at figure 2?

We thank the Reviewer for this constructive suggestion regarding the reproducibility of our pacing site selection. We agree that a purely manual selection can be perceived as subjective.

To clarify, the selection was guided by two primary criteria: spatial coverage and proximity to the infarct border. We prioritized sites that spanned the entire perimeter of the scarred region (e.g., covering the superior, inferior, and lateral sides). For each of these directions, we selected the pacing site (out of the 19 described) nearest to the border zone while ensuring it was not located within the non-conductive scar (the MI region). This approach ensured that the pacing stimulus was delivered to viable tissue capable of initiating reentrant pathways while maintaining a distribution that captured the heterogeneity of the border zone. We have updated Section 2.2.2 to explicitly state these criteria:

"To maintain computational efficiency while ensuring a representative induction study, a subset of at least 5 well distributed pacing sites (out of the 19 AHA-standardized locations) was selected for each model based on two primary criteria: spatial coverage and proximity to the BZ (Fig 2). Sites were selected to homogeneously span the MI perimeter, ensuring coverage across the superior, inferior, and lateral sides. For each anatomical direction, pacing sites closer to the BZ were prioritized, provided they were located within viable myocardium rather than within non-conductive scar tissue. This strategy ensured that the pacing stimulus was delivered to tissue capable of supporting reentrant activity while capturing the spatial heterogeneity of the proarrhythmic substrate and its response to localized triggers [5, 4]."

While we did not use a strict automated distance algorithm for this specific study, we have updated the manuscript to describe these selection criteria more formally to aid reproducibility. Furthermore, we have added two discussion points.

One discussion point provides evidence supporting our simplification decision in Section 4.2: "Furthermore, we adopted a more targeted distribution of pacing sites compared with the 19 locations (RV apex, RV outflow tract, and 17 AHA-based sites) evaluated by Arevalo et al. [2]. Our protocol

used a subset of at least 5 pacing sites selected from those 19 locations. Because the BZ constitutes the critical substrate for reentry [4, 5, 6], pacing sites were distributed homogeneously around the MI border, as more distant pacing often elicits redundant reentrant patterns. Consistent with this, a comprehensive AIP applied to the atria showed that electrophysiologically remodeled regions exhibit greater arrhythmia susceptibility and that a reduced number of homogeneously distributed pacing sites can capture up to 90% of induced arrhythmias compared with more exhaustive protocols [3]. Overall, these strategic simplifications enabled high-throughput assessment of the CS-BiV complex while preserving substantial computational scale, yielding reentrant patterns that closely resemble experimental observations (see Section 3.1)."

And another discussion point acknowledges that future automated approaches could further standardize the AIP. The complete "Limitations, uncertainties, and future work" text added for the AIP protocol is:

"***In silico* AIP:** The combination of a limited, low-resolution S2 range and a reduced number of pacing sites, rather than a high-resolution exploration of coupling intervals or a complete 19-site sweep, may fail to capture narrow spatiotemporal windows of vulnerability for VT induction. Consequently, models classified as non-inducible under our protocol could potentially exhibit reentry under a more exhaustive coupling-interval search. Thus, although our *in silico* AIP qualitatively reproduced subject-specific experimental VT patterns with substantially lower computational cost, future studies may benefit from more spatially detailed and temporally extended stimulation strategies. From a spatial standpoint, although no fully automated quantitative criteria were used for pacing-site selection, we implemented a reproducible strategy designed to homogeneously encircle the MI region and assess proarrhythmic BZ heterogeneity [5, 4, 3]. Future work could incorporate automated methods based on geodesic distance to scar tissue or the identification of slow-conducting isthmuses, as proposed by Campos et al. [8]. Temporally, broader and site-specific S2 coupling interval ranges could be examined using checkpointing strategies and automated estimation of the effective refractory period at each pacing site, following approaches similar to those described by Azzolin et al. [3]. Collectively, these methodological refinements would further standardize the AIP and enable systematic identification of the key spatial and temporal pacing features underlying arrhythmia induction, while maintaining computationally feasibility. Such advances may uncover reentry mechanisms that arise only within narrow temporal windows or specific trigger-substrate configurations."

Lines 146-148, please add explicitly the use of Meshlab with its corresponding reference. Also add the used method with the corresponding reference using the DOI provided in the Meshlab window of the function of the explicit remeshing method.

We thank the Reviewer for the suggestion to provide more technical detail regarding the mesh processing. We have updated the manuscript to explicitly mention the use of Meshlab and have included the specific references and DOI-linked methods for the XOR and isotropic remeshing filters.

The revised text in Section 2.3 now reads: "Following embedding, the EHT and BiV meshes were merged using an *XOR* operation in Meshlab [52, 53] (Fig 3c). The internal portion of the EHT located within the ventricular wall (blue region in Fig 3c) was removed. To ensure a high-quality volumetric mesh, irregular triangles at the BiV–EHT interface, which would otherwise lead to poor-quality tetrahedralization, were identified based on their aspect ratio and refined using the isotropic explicit remeshing filter in Meshlab [54, 53] (Fig 3d)."

Lines 152-156, one of my biggest concerns of this study is the decision of having a bimodal edge length distribution. The authors highlight in the limitation section the importance of the edge length by referencing the study of Bishop et al. However, the authors have not provided sufficient details on the impact of conduction velocity with the bimodal distribution of the edge length. In the EHT area of the model the same value of diffusion coefficient was used which I assume was calibrated for the 381.9 um mean edge length. This will result in a much higher conduction velocity in the EHT area as a mean resolution of 187.2 um. Please provide sufficient evidence that having two areas with very different resolutions will not impact the results of the study and this to the supplementary material of the study as this is a critical point for this work.

We thank the Reviewer for highlighting this critical point. We agree that non-uniform mesh dis-

cretization can introduce numerical artifacts if not carefully validated. In response to this concern, we have performed a comprehensive quantitative analysis of the simulated CVs across different regions and mesh resolutions to ensure that our bimodal distribution does not lead to non-physiological artifacts. Specifically, we performed a quantitative analysis of the CV and extended the discussion regarding mesh tetrahedralization density in the Section "Limitations, uncertainties, and future work".

Quantitative CV validation: We have added a new Section 2.6 ("Simulated conduction velocities"), where we calculated and compared CVs in the HZ, BZ, and EHT regions across all model configurations and against experimental, porcine-specific previously obtained OM measurements. To accomplish this, we conducted a sensitivity analysis on the CV calculation radius (now included in supporting figure S2 Figure) to ensure that the 1 ms resolution of activation times did not produce spurious results, especially in the fast conducting regions. We found that a 2 mm search radius provides biomimetic, finite CV values that are robust across both discretization scales. In this context, the new section and figures are:

"In Section 2.4, conductivity values for the CS and HZ were defined based on existing large-mammal literature (canine [34] and human [29]) and validated against our own porcine-specific experimental data (electrocardiogram and OM) obtained under sinus or paced rhythm (see our previous work [1]). Additionally, BZ conductivities were adjusted to reproduce post-MI conduction CVs previously measured via OM under sinus or paced rhythm [1]. In the present work, inclusion of the EHT modified the computational domain, requiring mesh refinement in the remuscularized region (see Section 2.3). To ensure that the selected conductivities, together with the non-uniform mesh resolution, preserved physiological accuracy, we evaluated the simulated CVs.

CVs were calculated across the epicardial faces of the BiV meshes to maintain consistency with the superficial nature of OM data [1]. The CV magnitude between two nodes was computed as the ratio of their Euclidean distance to the difference in their activation times. For each node, a mean CV vector was calculated within a defined radius [1]. As shown in S2 Figure, multiple radii were evaluated; smaller radii (e.g., 0.6 mm) produced numerical artifacts, specifically infinite CVs (red in S2 Figure), due to the 1 ms activation time resolution and the proximity of nodes with identical activation times, particularly in fast-conducting HZ. Conversely, larger radii yielded more biomimetic CVs in the HZ, with low variation upon further radial increments. Accordingly, a 2 mm radius was selected as the minimum threshold providing consistent, finite CV values in the HZ.

Under this configuration, CVs were computed for the final S1 stimulus in pig 6 (pacing site 15) across all modeling scenarios: the baseline MI model without EHT, and models with the EHT positioned at L1 and L2 under C10 and C90 conductivity settings (Fig 4). In the HZ, simulated CVs ranged from 69–79 cm/s. Despite differences in mesh resolution among cases, HZ values remained consistent and, notably, closely matched the experimental healthy porcine CV of 74.2 cm/s, previously measured by OM under sinus and paced rhythm [1]. Lower CV values were primarily attributable to the presence of the slower-conducting EHT (notably the L2-C10 configuration) rather than to variations in mesh edge length. Indeed, as shown in Fig 4a, CVs within the finely meshed EHT region were comparable to those of the underlying myocardium (BZ or HZ) and were consistent with the values obtained in more distant, coarser-meshed equivalent (BZ or HZ) regions. Similarly, CVs in the BZ were robust to changes in mesh resolution. Values were nearly identical (27 cm/s for C10; 34 cm/s for baseline MI and C90) regardless of whether the EHT was implanted directly above (L1), lateral to (L2), or absent from the region. These simulated CV values agree with the experimental porcine range of 27.9–43.6 cm/s measured in the MI region by OM under sinus and paced rhythms [1].

The CV in the EHT was influenced by both its intrinsic conductivity and the underlying myocardium, suggesting that the host tissue effectively drove EHT depolarization, as reported in [10, 12, 21]. At the L1 position with low conductivity (C10), the EHT exhibited a CV of 13.6 cm/s, consistent with reported *in vitro* EHT values (5–24 cm/s) [15, 14]. Because the EHT at this location predominantly overlies non-conducting SZ or slow-conducting BZ, the simulated CVs naturally approximated *in vitro* values. In contrast, when assigned adult-like conductivity (C90), the CV in the EHT increased, particularly at the L2 position where it overlies fast-conducting HZ, approaching healthy myocardial velocities.

Overall, this analysis confirms that the simulated CVs accurately reproduce porcine-specific experimental values under healthy and MI conditions, in both sinus and paced rhythms, as well as *in vitro* EHT dynamics, despite the non-uniform, bimodal mesh resolution. These results provide a solid

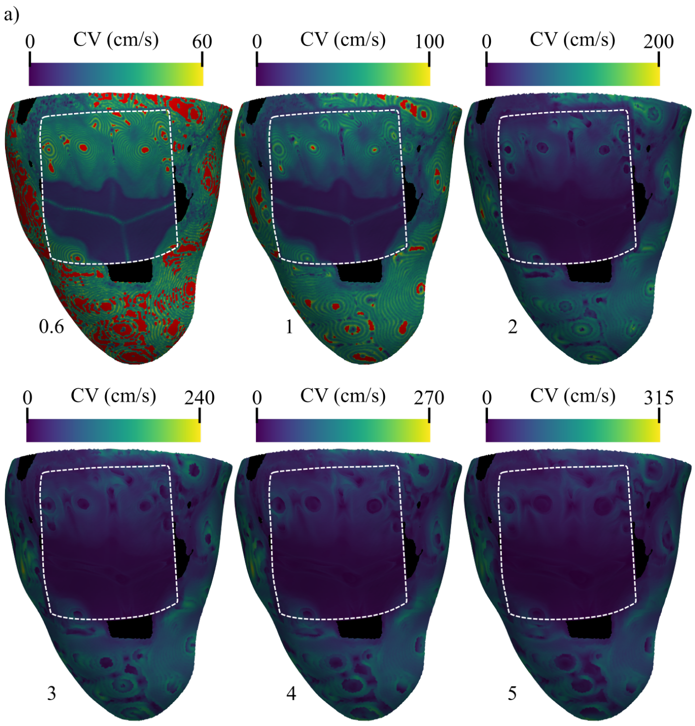

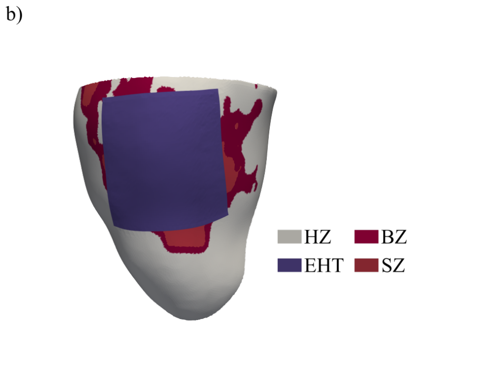

c)

Computation radius	median CV (cm/s) Healthy	Border zone	EHT
0.6	36.3	21.6	12.6
1	57.1	24.3	13.6
2	78.7	26.7	13.6
3	83.2	28.17	13.5
4	85	28.8	13.7
5	89.6	29	14

Figure 3: **Characterization of simulated conduction velocity (CV).** a- Node-wise CV computation was performed on the lateral surface of pig 6 following the last S1 stimulus at pacing site 15. The CV magnitude for a given node was calculated as the mean ratio of Euclidean distance to activation time difference relative to all neighboring nodes within a specified search radius. Sensitivity analysis of the search radius revealed that smaller radii yielded numerical artifacts (infinite values, shown in red) due to neighboring nodes sharing identical activation times (1 ms resolution). CV values in the fast-conducting healthy zone (HZ) stabilized as the radius increased. b- Anatomical segmentation of the lateral ventricular face, highlighting HZ, border zone (BZ), scar zone (SZ), and engineered heart tissue (EHT) regions. c- Median CV values calculated for the HZ, BZ, and EHT across the different search radii tested. A trade-off radius of 2 mm was selected for all subsequent calculations (highlighted in green), as it represented the minimum distance required to ensure numerical stability and convergence across all tissue zones.

foundation for our arrhythmia inducibility assessment. This quantitative accuracy is further supported by qualitative validation, in which simulated arrhythmic patterns were compared with subject-specific, OM-derived arrhythmic events (G1 simulations). Importantly, no additional adjustments to conductivity parameters were made using these OM data under arrhythmia, ensuring a clear separation between model calibration (based on OM of sinus and paced rhythms) and evaluation (based on OM of reentrant activity)."

In summary, these results demonstrate that CVs in the finely meshed EHT region are consistent with those in the coarser-meshed HZ/BZ regions. Specifically, as shown in Fig 4a, CV within the EHT region accurately reflects the properties of the underlying myocardium and aligns with both experimental porcine OM data and *in vitro* EHT benchmarks, independent of local mesh density.

Integration with recent literature: We have expanded the "Limitations, uncertainties, and future work" section to contextualize our modeling approach within recent computational studies on arrhythmia assessment, specifically the findings by Bishop et al. [55]. We clarify that the refined mesh density used for the EHT and adjacent regions ($\leq$ 200 µm) strictly adheres to the recommendations of Bishop et al. [55] to prevent spurious conduction block in low-CV zones. Moreover, we provide evidence that measured CVs remain aligned with experimental benchmarks across various tissues, even in regions with coarser grids, ensuring that our bimodal discretization does not introduce significant numerical artifacts. The revised discussion now reads:

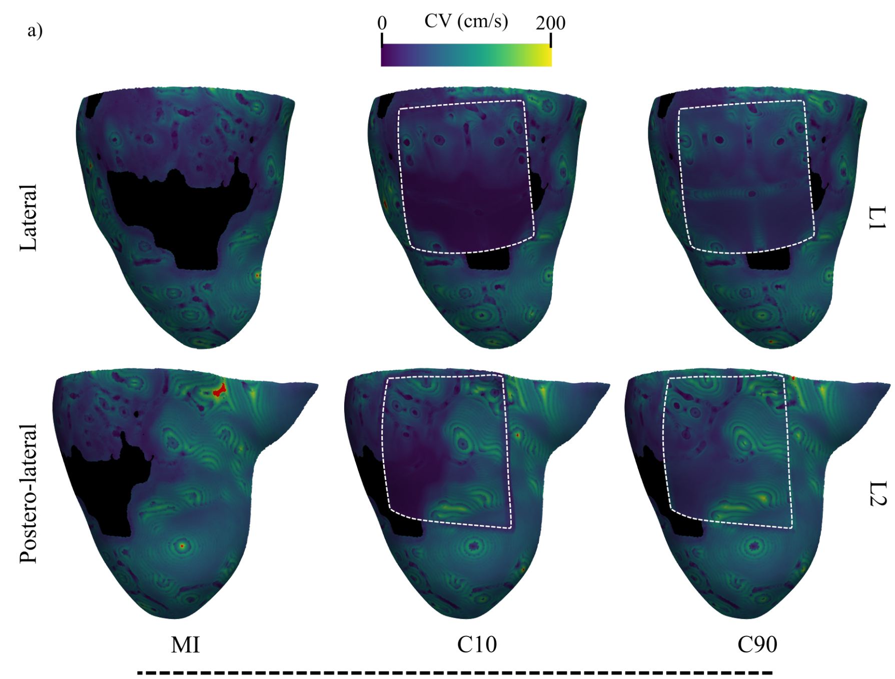

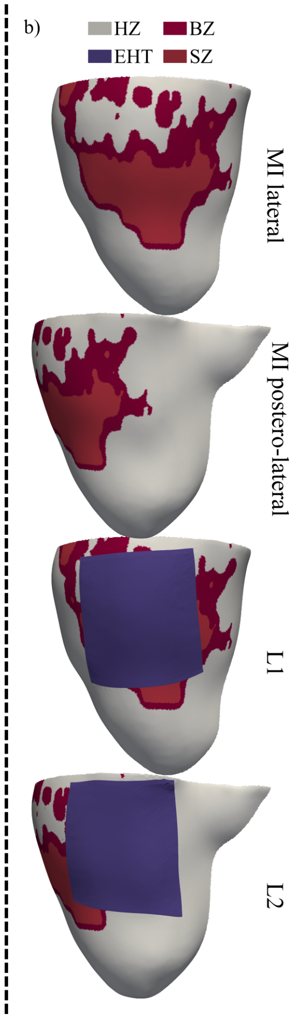

c)

Case	median CV (cm/s)		
	HZ	BZ	EHT
MI lateral	76.3	33.5	-
MI postero-lateral	73.6	33.8	-
L1-C10	78.7	26.7	13.6
L1-C90	79.2	34.1	40.8
L2-C10	69.4	27.1	38.5
L2-C90	72.4	33.7	56.2

Figure 4: **Simulated CV with and without the EHT.** a- Node-wise CV maps for pig 6 following the last S1 stimulus applied at pacing site 15 without the EHT (MI case) and when the EHT was implanted in different locations (L1-L2) and assigned different conductivities (C10-C90). Undefined values due to infinite CV (matching activation times among nodes) are depicted in red. b- Segmentation of the analyzed ventricular faces, highlighting HZ, BZ, and EHT regions. c- Median CVs obtained for different regions with and without the EHT.

"The tetrahedralization process employed edge lengths consistent with established literature [2, 33, 6, 56], which have been shown to reliably capture both physiological and arrhythmic dynamics. Specifically, Boyle et al. [57] demonstrated that edge lengths below 400 µm are sufficient to resolve reentrant circuits consistent with experimental observations while mitigating numerical conduction block artifacts. More recently, Bishop et al. [55] highlighted the need for finer discretizations ($\leq$ 200 µm) in low-CV regions to prevent spurious conduction block and reduce variability in arrhythmic outcomes. In our framework, median edge lengths below this 200 µm threshold were achieved within the EHT and adjacent regions. Furthermore, we applied the CV tuning strategies recommended by Bishop et al. [55] for coarser meshes: conductivities in infarcted regions were adjusted to match species-specific OM data obtained under sinus and paced rhythms [1], while settings in healthy and EHT regions produced CVs consistent with experimental benchmarks (see Section 2.6). Although coarser discretizations have been successfully used in prior studies [57, 56, 58, 10, 11, 2, 33], our multiscale meshing approach enhances the robustness of the inducibility assessment by combining high resolution in critical zones with validated conductivity selection."

We believe these additions provide the necessary evidence that the bimodal mesh resolution main-

tains physiological accuracy and does not negatively impact the assessment of arrhythmia inducibility.

Line 176-179, the authors provide diffusion coefficients which are useful but please add explicitly what is the corresponding conduction velocity that was achieved for Gaur et al. model and Paci et al. model. As mentioned before and by the authors the study of Bishop et al. shows the impact of resolution and and the choice of diffusion coefficients should be verified that reaches physiological conduction velocities that will result on numerical artificial block or in this case due to the different resolutions an acceleration of the propagation. Please add explicitly the values of conduction velocities used in this study.

We agree with the Reviewer that diffusion coefficients alone do not fully describe the electrophysiological behavior, particularly across varying mesh resolutions. To address this, we have explicitly calculated and reported the resulting CVs for all tissue components in the revised manuscript.

As requested, the CV values resulting from the integration of the Gaur et al. [9] (myocardial) and Paci et al. [23] (EHT) ionic models with our chosen conductivity profiles are now detailed in a novel Section 2.6, entitled "Simulated conduction velocities" (see previous answer), and summarized in Fig 4c. Specifically:

- Healthy Myocardium: The conductivity definition (utilizing an LDC of 0.0013 cm^2/ms, a transverse-to-longitudinal ratio of 0.25, and a ±60° standard fiber alignment) yielded a median epicardial CV of 69–79 cm/s. This closely matches our porcine-specific experimental benchmark of 74.2 cm/s obtained via OM during sinus and paced rhythms in our previous work [1].
- Infarct Border Zone: The conductivity definition (utilizing an LDC of 0.000882 cm^2/ms, a transverse-to-longitudinal ratio of 0.345, I_{Na} reduced to 38% of its default value, and a ±60° standard fiber alignment) yielded a CV of 26.7–34.1 cm/s. These values are consistent with the experimental post-MI range of 27.9–43.6 cm/s obtained from subject-specific OM measurements in LAD-infarcted pigs during sinus and paced rhythms [1].
- Engineered Heart Tissue: For the low conductivity profile (C10: LDC of 0.00013 cm^2/ms, transverse-to-longitudinal ratio of 1) at location L1, where minimal contact exists between the EHT and the underlying conducting tissue (BZ and HZ), the resulting CV was 13.6 cm/s, aligning with reported *in vitro* values (5–24 cm/s). For the adult-like conductivity profile (C90: LDC of 0.00117 cm^2/ms, transverse-to-longitudinal ratio of 1), the CV in the EHT reached its peak (56.2 cm/s) when in contact with fast-conducting HZ, reflecting how the host myocardium accelerates EHT depolarization (or, alternatively, contains it when the EHT is in contact with the BZ).

Importantly, our analysis in Section 2.6 demonstrates that the conductivity profiles employed did not cause numerical acceleration or spurious conduction block. Instead, they produced physiological, species-specific, and consistent CVs across all tissue regions, despite the use of non-uniform, fine mesh resolutions.

Line 184, the authors mention a "physiological anterograde and retrograde electrical 184 propagation between CS and ventricular tissue". Please provide the evidence to support this. There should be a delay in the retrograde propagation activation. Was this considered in this work? If this was also considered please add it to the manuscript or comment on the limitation section as this will be a key aspect on the impact of the CS results shown in this study.

We thank the Reviewer for this comment. We agree that the bidirectional conduction properties and the associated delays at the PMJs are critical factors in the accuracy of cardiac simulations.

In this work, bidirectional conduction was achieved by implementing a sigmoid-based reduction of the conductivity at the CS endbranches, following the methodology proposed by Dux-Santoy et al. [31]. To clarify this, we revised the text in Section 2.4, which now reads:

"The LDC for the CS was reduced near its endpoints using a sigmoid profile to match the ventricular LDC, as proposed by Dux-Santoy et al. [31], thereby enabling retrograde propagation at the PMJs. Bidirectional conduction across the PMJs was achieved (see S1 Figure). A minimal anterograde

propagation delay of 1.1 ms was observed, whereas the retrograde delay was zero (see discussion in S1 Section and Section 4.6)."

Regarding the Reviewer's comment about the delays:

- Bidirectional Modeling: We explicitly quantified these delays in our porcine CS-BiV model. We found an anterograde delay of 1.1 ms and a retrograde delay that was negligible (<1 ms) within the temporal resolution of our simulation.
- Physiological Justification: The observed asymmetry (anterograde delay > retrograde delay) is consistent with the well-established source-sink mismatch principle [59, 60, 1]. In pigs, this effect is further accentuated because PMJs are located deeper within the intramural myocardium compared to canine or human models [46, 61], interfacing with a larger myocardial mass that facilitates retrograde capture while resisting anterograde spread [1].
- Experimental Context: While classical canine studies report slightly higher values (2–4 ms), those measurements are often limited by a spatial resolution (≈1 mm) that cannot resolve the sub-millimetric distances (0.5 mm) analyzed in our PMJs.

To address this in the manuscript, in addition to rephrasing the mentioned text in Section 2.4, we added a detailed characterization of these delays in the supporting material. The new S1 Section, entitled "Delays of anterograde-retrograde propagation at the PMJ", and the S1 Figure are:

"As shown in S1 Figure, both anterograde and retrograde electrical propagation across the PMJs was successfully captured. To quantify direction-dependent propagation delays, a localized region of the CS-BiV complex near pacing site 15 (pig 6) was analyzed following the final S1 stimulus. The propagation delay at a PMJ was defined as the mean activation time difference between the CS endpoint and its associated myocardial nodes. As defined in Section 2.1, these myocardial nodes were identified as ventricular nodes within a 0.5 mm radius of the CS endpoint. Across the full CS-BiV domain, complex phenomena can introduce confounding effects that preclude a clean calculation. These include the simultaneous depolarization of a PMJ from both the CS and myocardial tissue, as well as variations in local PMJ density that alter the source–sink mismatch and modify conduction delays by changing electrotonic loading effects [60]. Consequently, the analysis was restricted to a reduced postero-apical region near the pacing site, focusing specifically on the three PMJ propagation events highlighted in S1 Figure.

As illustrated in S1 Figure, three epicardial breakthroughs are observed: one (red star in S1 Figure) originating from stimulation at endocardial pacing site 15, producing a retrograde reentry, and two (black stars in S1 Figure) resulting from anterograde depolarization through distinct PMJs. The retrograde delay was zero, suggesting that the activation of the CS endpoint occurred simultaneously with that of the connected myocardial nodes. However, this measurement was limited by the temporal resolution of our simulations (1 ms). For anterograde conduction, a mean delay of 1.1 ms was measured across the two PMJs propagating in the anterograde direction.

Computational studies of bidirectional conduction have calibrated higher delay values, particularly in human-oriented modeling and simulation, largely based on canine experimental data. For example, Gillette et al. [49] used anterograde and retrograde propagation delays of 8 and 3 ms, respectively. In closer agreement with the present work, Riebel et al. [19] used delays of 2 and 1 ms for anterograde and retrograde propagation. Experimentally, Veenstra et al. [62] reported anterograde delays of 2–4 ms in canine studies and reviewed evidence suggesting that anterograde delays below 5 ms are more physiological than larger values. Retrograde delays are consistently reported to be shorter than anterograde delays, reflecting faster myocardium-to-CS conduction, likely due to the greater electrotonic load imposed on the CS endpoint by the bulkier myocardial tissue, a source–sink mismatch widely observed *in silico* [63, 59, 1]. We previously showed that this effect is accentuated in pigs [1], as PMJs are located deeper within the myocardium than in dogs and humans and contact a larger myocardial mass for a given PMJ size [46, 61], thereby supporting the lower retrograde delay values obtained in the present study. Under this hypothesis, larger anterograde delays than those reported experimentally in dogs would also be expected. Indeed, when myocardial nodes located farther from the CS endpoint are considered in the anterograde delay calculation, higher values are obtained (mean anterograde delays of 1.7 and 2.7 ms for myocardial nodes located up to 0.6 and 1 mm from the CS

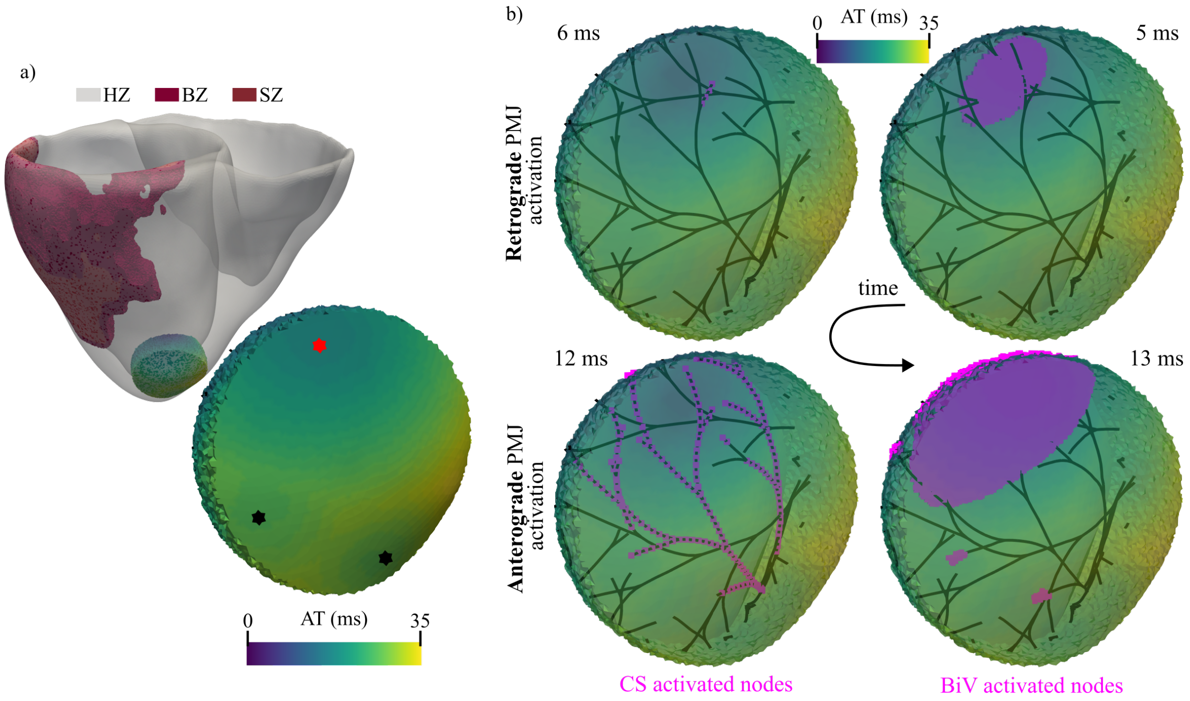

Figure 5: **Characterization of PMJ anterograde and retrograde propagation delays.** a- Analysis of the postero-apical region (pig 6) following the last S1 stimulus at pacing site 15. Three distinct epicardial breakthroughs are observed, arising from the interaction between endocardial stimulation and CS depolarization. b- The endocardial stimulus applied at 0 ms (snapshot at 5 ms, top-right) triggers retrograde propagation at a PMJ (CS node ID 2350) at 6 ms (top-left), producing the first epicardial breakthrough (red star, panel a). This wavefront propagates rapidly through the CS (12 ms, bottom-left), culminating in two anterograde PMJ activations at 13 ms (bottom-right, CS node IDs 1592 and 17346), which manifest as the two secondary breakthroughs (black stars, panel a).

endpoint, respectively). This observation is particularly relevant given that the experimental measurements used were inherently limited in spatial resolution and are typically unable to resolve distances smaller than 1 mm [62].

Taken together, we contend that the anterograde and retrograde delays employed here are consistent with physiological values: anterograde delays are systematically greater than retrograde delays, and the retrograde delay can reasonably be assumed to be zero due to the increased source–sink mismatch associated with the deeper CS in pigs. Future studies may further refine these parameters by incorporating porcine-specific experimental data, which are currently lacking."

Lastly, we added the notion that novel porcine-specific data is needed for further validation or calibration of our simulation study. The modified text in Section 4.6 is:

"As additional data become available, porcine-specific functional (e.g., ionic dynamics, conductivity, and anterograde-retrograde PMJ delays) and structural (architecture, PMJ location) CS features should be integrated into the modeling framework to enable more robust preclinical modeling of CS influence on depolarization and arrhythmia."

Line 191, add explicitly how the insulator was modeled in the monodomain simulations of this study.

We thank the Reviewer for the opportunity to clarify this technical implementation.

In the monodomain framework, modeling the scar zone as an insulator requires the enforcement of a zero-flux boundary condition ($\mathbf{n} \cdot (\sigma \nabla V_m) = 0$) at the interface between the conducting tissue and the scar. In this study, this was achieved by physically removing the tetrahedrons that constitute the SZ from the computational domain, effectively creating a no-flux boundary at the excised surfaces.

We have updated the manuscript to provide a detailed description of this process, specifically

highlighting how we handled "mixed" elements to preserve narrow conducting isthmuses that are critical for reentrant dynamics. The revised text in Section 2.4 now reads:

"As transmembrane potential (V_m) gradients have not been observed in experimental chronic MI electrocardiograms [1, 33], the SZ was modeled as an insulator. This was implemented by establishing a zero-flux boundary condition around the SZ by simply excising it, since a Neumann boundary condition is used in the monodomain model used herein (see Section 2.5). Specifically, all tetrahedra composed exclusively of SZ nodes were removed from the computational domain. For the remaining tetrahedra containing at least one SZ node, those nodes were retagged as the non-SZ nodes within the same tetrahedron. This approach was adopted because removing all elements containing any SZ node would be overly aggressive and could eliminate narrow, low-conducting BZ isthmuses, which are critical substrates for reentrant tachycardias [5, 33, 35, 8]."

Lines 194-199, please add the number of pulses needed for each model to reach the limit cycle. For the Stewart et al. model there is a comment on the automaticity. How about the automaticity of the Paci et al. model? Was there also a stabilization done on the 3D geometry?

We thank the Reviewer for this suggestion. We have updated Section 2.4 to explicitly state the number of pulses (beats) required to reach the limit cycle (steady state) for each cellular model. Additionally, we have clarified our approach regarding the automaticity of the Paci et al. [23] model and provided the physiological rationale for the prepacing protocol. The updated paragraph in Section 2.4 now reads:

"All ventricular cell models were prepaced to steady state by applying periodic S1 stimuli (80 $\mu A/cm^2$ magnitude, 1 ms duration, 600 ms period). For the Paci et al. [23] model, prepacing was performed according to the stimulation described in [23] (0.55 nA magnitude, 5 ms duration) with a period of 600 ms. The intrinsic automaticity of the Paci et al. [23] model was preserved without modification. Stimulated prepacing was used to reflect standard preculturing protocols, in which external electrical stimulation is applied to enhance hiPSC-CM maturation [64]. Stabilization of all cellular models was confirmed by verifying that APD_{90} remained constant across consecutive beats. Although repolarization reached a steady state after 800 s, a total of 2000 beats (1200 s simulation time) were simulated to ensure final convergence of APD_{90} to: 310 ms for the CS (under spontaneous automaticity), 196-218 ms across all transmural and apicobasal regions of the HZ, 384 ms for the BZ, and 405 ms for the EHT."

Regarding the three-dimensional geometry, spatial stabilization and the establishment of a reproducible depolarization-repolarization sequence were achieved through the four S1 stimuli applied at the beginning of our arrhythmia inducibility protocol. This ensured that the tissue-level electrophysiology was stabilized prior to the introduction of the S2 trigger.

Lines 239-240, is this claim valid for this context of cardiac electrophysiology simulation on pigs? Nowadays, most of the studies that use an HPC system are computing millions of seconds of simulated time. Please comment on this claim specially to current studies.

We thank the Reviewer for this constructive suggestion. We have revised the text to more precisely characterize the computational scale and technical advancements of our study. Specifically, we now emphasize that the cumulative simulated time, coupled with high mesh resolution and the incorporation of multi-scale electrophysiological heterogeneities, aligns this work with current cutting-edge research, such as the studies by Yu et al. [10] and Riebel et al. [19]. Furthermore, we have clarified that our modeling of the EHT-myocardium interface represents a significant advancement in anatomical and physiological realism for organ-scale simulations, particularly regarding the complex graft-host interaction. The revised ending of Section 2.5 is:

"In total, 335 simulations were performed, representing a cumulative duration of more than one million milliseconds of electrophysiological activity. Although large-scale simulated time is increasingly common in HPC-based cardiac research, the scale of this study is particularly notable given the high spatial resolution of the BiV meshes and the dense incorporation of electrophysiological heterogeneities. The substantial wall-clock time required and the fine spatial discretization place this work at the forefront of current simulation studies on remuscularization [10, 11, 19]. Furthermore, by modeling the EHT as directly implanted onto the epicardial surface of the BiV anatomy, we introduce an

enhanced level of structural and physiological realism. This detailed representation of the graft-host interface at the organ scale captures a level of complexity not previously addressed in simulations of EHT-mediated remuscularization [10, 11], thereby providing a more robust framework for evaluating post-implantation arrhythmogenicity."

Subsection 3.3, the authors argue that the CS can either sustain VT (via bypasses and "breakthroughs") or suppress VT, and reports that removing CS decreases IS in HI pigs but increases IS in LI pigs. While this is plausible and aligns with the notion that Purkinje/PMJ pathways can change reentry dynamics, the presented evidence reads mainly as case-based. Please add quantitative descriptors of the mechanism (e.g., number/location of breakthroughs, timing of PMJ activation relative to wavefront arrival, changes in conduction time across regions, rotor period/trajectory statistics, Vm traces, etc) to substantiate the interpretation beyond visual inspection of figures 6 and 7. Additionally, explain the choice of the analysis of figure 8. Why not do the same analysis as in figure 5?

We thank the Reviewer for this insightful comment. In light of our findings, we consider that the CS can either facilitate or hinder VT through the electrical bypasses they create between distinct myocardial regions. These conduction shortcuts may produce breakthroughs and/or modulate the depolarization of the slow-conducting, long-APD MI region. In turn, this can initiate an MI-derived depolarizing wave that sustains the depolarization–repolarization in the non-refractory HZ.

We agree with the Reviewer that these findings are case-based. Accordingly, we have revised the final paragraph of this section to explicitly state that our conclusions apply to the specific samples analyzed and may not be universally generalizable. Nevertheless, even as case-based observations, these results support the need for accurate subject-specific modeling of the CS to reliably assessing arrhythmic risk. Although related concepts were already discussed in Section 4.3, we have now clarified and reinforced this point in Section 3.3. The revised ending of Section 3.3 now reads:

"These results suggest that the electrical bypasses provided by the CS can generate breakthroughs or modulate the activation of the slow-conducting, long-APD regions, leading to subsequent reactivation of the surrounding non-refractory tissue, thereby either sustaining or impeding VT. Consequently, although case-based, these findings highlight the importance of accurately modeling the CS to ensure a robust and reliable evaluation of ventricular arrhythmogenesis."

Regarding the inclusion of quantitative descriptors of the underlying mechanisms, many of these approaches require the development of complex algorithms and extensive validation to ensure accurate and reliable quantitative characterization of the arrhythmia. Such efforts are beyond the scope of the present study. Accordingly, we have added a statement in Section 4.3 outlining future research directions, in which more advanced quantitative algorithms could be implemented to further support our observations and expand the mechanistic analysis presented herein:

"Next, more advanced quantitative descriptors [63, 59] - including breakthrough counting and spatial localization analysis; rotor-related metrics such as period and trajectory; phase singularity computation; and quantification of successful versus failed anterograde and retrograde propagation across PMJs - may be systematically incorporated to further elucidate the mechanistic insights introduced herein, while enabling a more rigorous and quantitative assessment of VT complexity by discriminating among anatomically anchored reentry, functional rotor dynamics, and PMJ-mediated propagation mechanisms."

Nevertheless, we agree with the Reviewer that additional data can help substantiate the findings presented in Section 3.3. Therefore, we have included additional V_m traces at strategically selected locations within the simulations presented in Section 3.3 to further support our results. The revised text in Section 3.3 now reads:

"For the group of LCx pigs:

- *Pig 6:* Exemplified a case of low inducibility in which CS played a fundamental role in blocking the reentry. As shown in the first row of Fig 6, application of AIP at pacing site 6 generated an S2-derived depolarization wave that initially propagated from the anterior to the posterior LV via the apex. When the CS was present, a CS-derived epicardial breakthrough intercepted this S2-derived activation in the laterobasal region, effectively preventing reentry. In contrast, without the CS, the same S2-derived activation encountered no such breakthrough, allowing sVT to develop. This outcome is further corroborated by the V_m traces recorded after the final S1

stimulus (see right column of Fig 6). With CS, the laterobasal (anterior: purple AP; posterior: yellow AP) and apical (green AP) regions were activated almost simultaneously by the last S1 stimulus compared with the condition without CS. In this scenario, the anterior laterobasal region (purple) remained refractory upon the arrival of the S2-derived wavefront, resulting in a unidirectional block. Without CS, this led to counter-clockwise reentry, as evidenced by the sequential depolarization observed in the green, yellow, and purple V_m traces. With CS, however, an epicardial breakthrough caused simultaneous depolarization of the anterior (purple) and posterior (yellow) laterobasal regions, thereby blocking reentry.

- *Pig 7:* This pig was selected as an example of high inducibility (pig 5 was not selected because nsVT in pig 5 propagated through the low-conducting laterobasal isthmus in the LV and might disappear in a whole-heart simulation, as described in Section 3.6). The application of AIP at pacing site 17 of pig 7 resulted in nsVT in the simulation with CS and in NR in the simulation without CS. In the former case, the CS enabled the MI to function as a capacitor: the faster depolarization and subsequent repolarization of the MI region permitted gradual loading of this region with S2-derived activation, followed by the release of the capacitive energy stored in the MI region from the apex back to the HZ once the refractory-based impedance in the HZ was low enough to allow the MI to be discharged, thereby producing reentry. Without CS, the S2-derived activation did not penetrate the MI region, which remained refractory. As shown by the corresponding V_m traces, the CS promoted more synchronous S1-induced activation of the MI (purple AP) and apical (green AP) regions. This synchronization allowed the S2-induced depolarization to reach a less refractory MI region (see V_m traces at 390 and 490 ms). Consequently, the MI became activated and sustained depolarization, as evidenced by the S2-derived apical-MI-apical activation sequence observed in the V_m trace with CS, which was absent without CS.

Similarly to the LCx cases, the presence or absence of CS determined an inversion in reentry occurrence in LAD pigs:

- *Pig 11:* Associated with HI among LAD pigs, the CS initiated delayed endocardial activations when pacing was applied at site 7. These activations propagated transmurally, producing epicardial breakthroughs at 590 and 840 ms after the last S1 stimulus, thereby sustaining VT (see the V_m maps and local traces in the third row of Fig 6). These epicardial breakthroughs are further supported by the higher activation rate observed in the anterobasal region (purple AP, third row of Fig 6) compared with the apical region (green AP, third row of Fig 6). Beyond its function as an *electrical bypass*, the CS also influenced VT dynamics through other mechanisms linked to its intrinsic cellular properties. A weak, initially undeveloped depolarization wavefront was reenergized in regions adjacent to the PMJs and the MI (see Fig 7b, which illustrates this phenomenon for the S2 stimulus applied at pacing site 4 in pig 6). During diastole, the CS exhibited a slightly higher resting potential than the surrounding myocardium, elevating V_m in nearby ventricular PMJ nodes (Fig 7a). Moreover, because the CS had a shorter APD than the BZ (Section 2.4), myocardial tissue surrounding the MI displayed reduced APD compared with the condition without CS. As illustrated in Fig 7c, V_m traces from the HZ near the MI and BZ showed shorter APDs in the presence of CS. Additionally, stimulation of the PMJs, even when originating from weakly depolarizing myocardial nodes, can suffice to trigger PMJ activation, given the higher excitability of the CS relative to the myocardium. This, in turn, reinforces the initially weak depolarization of the surrounding myocardial tissue. Collectively, these mechanisms suggest a facilitated propagation of depolarization in the presence of CS, thereby promoting VT sustainability. In contrast, without CS, reenergization of weak depolarization wavefronts and, more importantly, *electrical bypass*-mediated epicardial breakthroughs were absent (see the third row of Fig 6), and VT became non-sustained, terminating approximately 1 s after the last S1 stimulus. As illustrated in the corresponding V_m traces, depolarization of the apical region was inhibited because the tissue remained refractory, as evidenced by the final undeveloped AP in the green V_m trace.

- *Pig 12:* Exhibiting the lowest inducibility among LAD pigs, the presence of CS created an *electrical bypass* between the septal base and the apex of both ventricles when AIP was applied at the septal base (pacing site 3). The V_m traces of the septal base (purple AP) and apical region (green AP) began almost simultaneously in the presence of CS. This bypass enabled

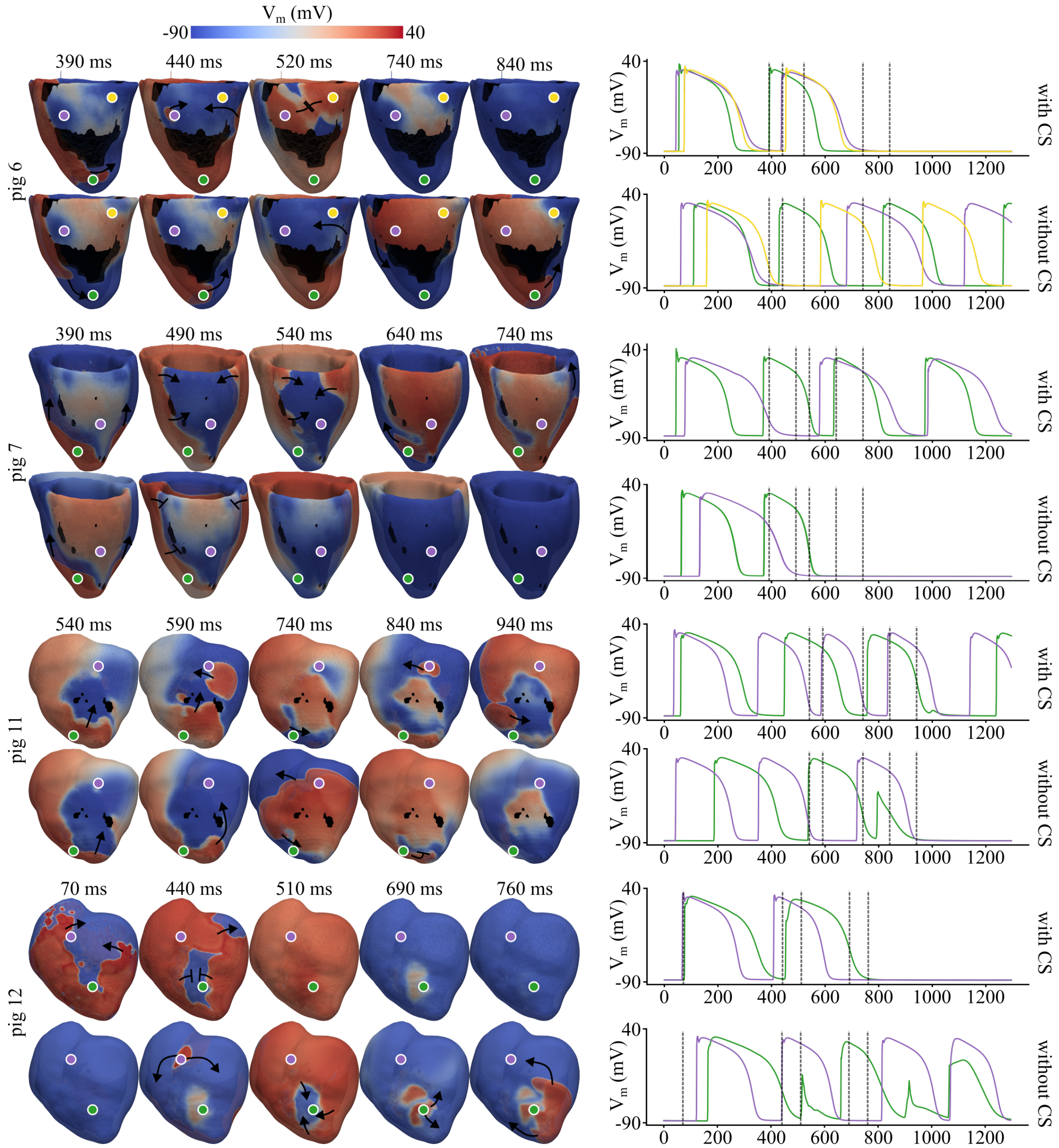

Figure 6: **Changes in proarrhythmicity associated with CS (G2 simulations)**. V_m maps and corresponding local traces are shown in the left and right columns, respectively. Results are presented for pig 6 (LCx LI) stimulated at pacing site 6, pig 7 (LCx HI) at pacing site 17, pig 12 (LAD LI) at pacing site 3, and pig 11 (LAD HI) at pacing site 7, displayed in the first through fourth rows, respectively. Time is referenced to the application of the last S1 stimulus; accordingly, the premature S2 stimulus was delivered at 295 ms in all cases shown. V_m traces were recorded at the following node IDs: pig 6 - 1923820 (green), 259964 (purple), and 1249245 (yellow); pig 7 - 564196 (green) and 1528977 (purple); pig 11 - 1638582 (green) and 3590756 (purple); and pig 12 - 2597575 (green) and 3205945 (purple). The vertical, dashed black lines in the V_m traces correspond to the time points of the V_m maps.

earlier depolarization of the MI region compared with the condition without CS (see the bottom row of Fig 6). Consequently, the S2 stimulus arrived at a fully repolarized MI 440 ms after the last S1 stimulus, resulting in NR. Without CS, a *capacitive effect* similar to that described for pig 7 with CS was observed. The S2-derived depolarization wave partially stalled within the

MI and gradually penetrated this region. From the V_m traces and the supporting S2 Video, the complex reentrant activity in this case can be appreciated. The apical MI region (green AP) initially exhibited incomplete, transient depolarizations before eventually undergoing full depolarization, as reflected by the transition from undeveloped to fully developed V_m upstrokes. Reentry subsequently occurred when the energy temporarily stored in the apical MI was released back into the HZ once excitability was restored. This initial reentrant event triggered a complex, polymorphic reentrant pattern that continued to sustain VT (see S2 Video)."

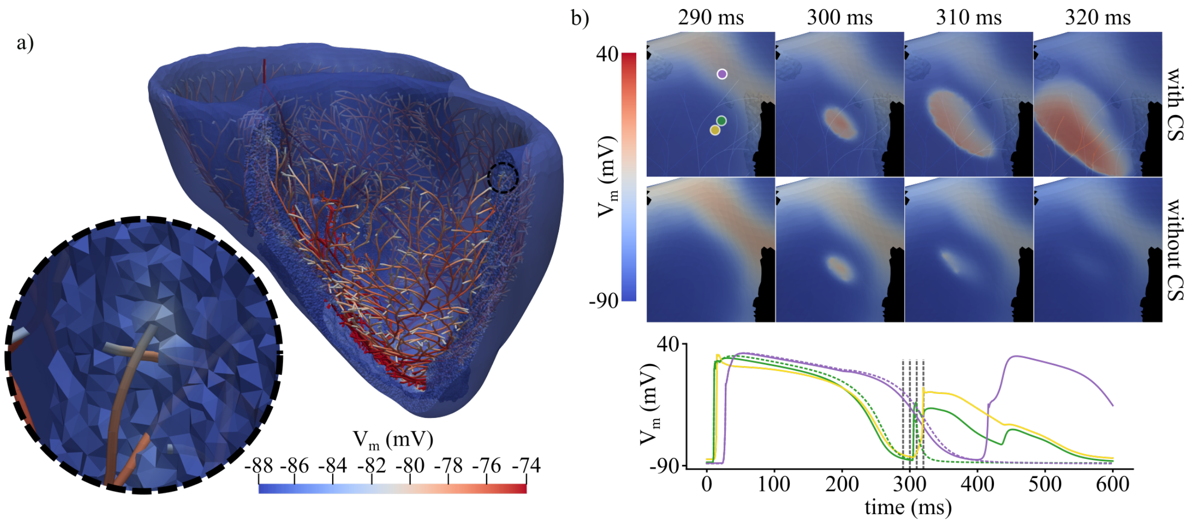

Figure 7: **Effect of CS intrinsic cellular properties.** a- Diastolic potential difference between CS and BiV domain of pig 11, before applying the fourth S1 stimulus at pacing site 7. b- S2-stimulus propagation when applied at pacing site 4 in pig 6 with and without the CS. V_m maps (top) and local traces (bottom) are depicted. V_m traces were obtained at myocardial nodes in the HZ (ID 1576842, green waves) and BZ (ID 576376, purple waves) when the CS was present (solid lines) or absent (dashed lines), and for the PMJ (CS endpoint ID 5873, yellow wave) near the activation. The vertical, dashed black lines correspond to the time points of the V_m maps at the top.

Finally, the results presented in Section 3.3 were previously summarized in Fig 8 (now incorporated into Fig 8, Fig 9 in the revised manuscript) in terms of changes in the inducibility score associated with the presence or absence of CS, as this metric provided a clear synthesis of our observations. Following the Reviewer's suggestion, we have now included the VT inducibility in the revised Fig 8, offering a more detailed representation of the outcomes for each individual simulation. The revised final paragraph of this section, reflecting this extended analysis, now reads:

"In general, the presence or absence of CS markedly influenced arrhythmogenesis. Notably, in two cases the S2 stimulus failed to capture in the absence of CS (see VT inducibility in the left panel of Fig 8). Mechanistically, this loss of capture was attributable to the absence of PMJ-mediated reenergization of a weak depolarizing wavefront, as previously described (Fig 7b). As shown in the right panel of Fig 8, removal of CS decreased IS in the HI cases for both MI types (from 0.33 to 0.11 in LCx and from 0.66 to 0.33 in LAD pigs), but increased IS in LI cases (from 0 to 0.25 in LCx and from 0.22 to 0.44 in LAD pigs). These findings suggest that the electrical bypasses provided by the CS can generate breakthroughs or modulate activation of slow-conducting, long-APD regions, leading to subsequent reactivation of the surrounding non-refractory tissue and thereby either sustaining or impeding VT. Consequently, although case-based, these findings highlight the importance of accurately modeling the CS to ensure a robust and reliable evaluation of ventricular arrhythmogenesis."

Subsection 3.5, the author shows the results of the states of maturation implemented via changing EHT conductivity and shows that increasing to C90 can suppress VT in some LCx cases (group IS 0.5→0.25) but not LAD (IS stable at 0.57). However, the issue of conduction velocity appears again here. Please check my comments above regarding resolution and conduction velocity. I would suggest changing "Contribution of the ETH maturation" for "Contribution of the ETH electrical coupling" as

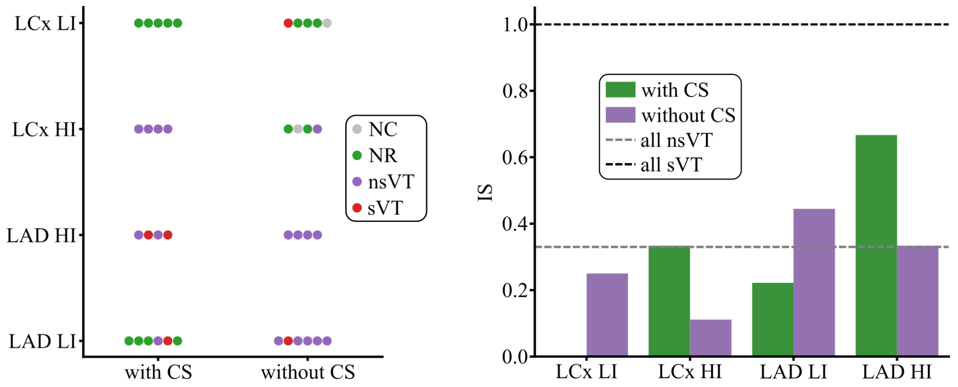

Figure 8: **Effect of CS presence or absence on inducibility (G2 simulations).** VT inducibility (left) and IS (right) computed with and without CS for LI and HI pigs of each MI type (LAD, LCx). LCx LI: pig 6, LCx HI: pig 7, LAD LI: pig 12 and LAD HI: pig 11.

other aspects of maturation are not explored. As commented before maturation is a more complex aspect that should also affect the APD of the cells in the ETH patch. The authors do not comment on the effect of the Paci et al. model. This model has a long APD90 and automaticity. When coupling the ETH path to the ventricular geometry how are cellular properties changed? How will these different cellular properties impact the suppression of arrhythmias? Was the automaticity of the Paci et al. model suppressed?

We thank the Reviewer for this valuable comment.

Conductivities, CVs, and mesh resolution: In the revised manuscript, we have added a justification for the selected low and high conductivity values and clarified how these parameters were previously validated against experimental measurements. As noted in our earlier responses, we have also incorporated a new section entitled "Simulated conduction velocities", in which we corroborate the attainment of physiological CV values consistent with experimental OM measurements (performed in previous works [1, 15, 14, 22]) in both the myocardium and the EHT. Furthermore, we have expanded Section 4.6 to further discuss the impact of mesh resolution, emphasizing the importance of CV calibration across different discretization levels. We also reinforced that the conductivity values used in this study were validated through direct comparison between *in silico* and experimentally measured CVs in our prior work [1, 15, 14, 22]. Collectively, these additions strengthen the robustness of our modeling framework, particularly with respect to conductivity parameterization across domains with varying discretization strategies. The revised text now reads:

- Low EHT conductivity (Section 2.5): "In all G3 simulations, the conductivity for immature EHTs was set to 10% (C10) of the value found in the HZ (0.00013 cm^2/ms) to match experimental observations. This value was previously validated in studies where simulated maximum activation times and CVs (≈12 cm/s) closely matched OM measurements from *in vitro* EHTs [22, 15]. Experimentally measured velocities typically range from 5 to 24 cm/s depending on scaffold architecture and EHT culturing [15, 14]."

- High EHT conductivity (Section 2.5): "Setting the S2 stimulus at 295 ms for this pacing site, all coupled BiV-EHT models, with EHT located at L1 and L2, were evaluated with the EHT conductivity set to 90% (C90) of the HZ value. This conductivity represents a mature, highly integrated scenario. Computational evidence indicates that matching the host myocardium's conductivity is critical to prevent the EHT from acting as a proarrhythmic substrate [20, 10, 11, 22, 12, 21]. Such high functional maturation is increasingly achievable through advanced scaffold designs, including diamond-shaped pores for directional stiffness control or the incorporation of electroconductive materials, which enhance cell alignment, connexin-43 expression, and electrical integration [15, 36, 37]. Consequently, results from C10 (immature, low conductivity) and C90 (mature, high conductivity) EHTs were compared for each subject to quantify the effect of post-implantation electrical maturation on overall VT risk."

- Novel Section 2.6: "In Section 2.4, conductivity values for the CS and HZ were defined based on existing large-mammal literature (canine [34] and human [29]) and validated against our own porcine-specific experimental data (electrocardiogram and OM) obtained under sinus or paced rhythm (see our previous work [1]). Additionally, BZ conductivities were adjusted to reproduce post-MI conduction CVs previously measured via OM under sinus or paced rhythm [1]. In the present work, inclusion of the EHT modified the computational domain, requiring mesh refinement in the remuscularized region (see Section 2.3). To ensure that the selected conductivities, together with the non-uniform mesh resolution, preserved physiological accuracy, we evaluated the simulated CVs.

  CVs were calculated across the epicardial faces of the BiV meshes to maintain consistency with the superficial nature of OM data [1]. The CV magnitude between two nodes was computed as the ratio of their Euclidean distance to the difference in their activation times. For each node, a mean CV vector was calculated within a defined radius [1]. As shown in S2 Figure, multiple radii were evaluated; smaller radii (e.g., 0.6 mm) produced numerical artifacts, specifically infinite CVs (red in S2 Figure), due to the 1 ms activation time resolution and the proximity of nodes with identical activation times, particularly in fast-conducting HZ. Conversely, larger radii yielded more biomimetic CVs in the HZ, with low variation upon further radial increments. Accordingly, a 2 mm radius was selected as the minimum threshold providing consistent, finite CV values in the HZ.

  Under this configuration, CVs were computed for the final S1 stimulus in pig 6 (pacing site 15) across all modeling scenarios: the baseline MI model without EHT, and models with the EHT positioned at L1 and L2 under C10 and C90 conductivity settings (Fig 4). In the HZ, simulated CVs ranged from 69–79 cm/s. Despite differences in mesh resolution among cases, HZ values remained consistent and, notably, closely matched the experimental healthy porcine CV of 74.2 cm/s, previously measured by OM under sinus and paced rhythm [1]. Lower CV values were primarily attributable to the presence of the slower-conducting EHT (notably the L2-C10 configuration) rather than to variations in mesh edge length. Indeed, as shown in Fig 4a, CVs within the finely meshed EHT region were comparable to those of the underlying myocardium (BZ or HZ) and were consistent with the values obtained in more distant, coarser-meshed equivalent (BZ or HZ) regions. Similarly, CVs in the BZ were robust to changes in mesh resolution. Values were nearly identical (27 cm/s for C10; 34 cm/s for baseline MI and C90) regardless of whether the EHT was implanted directly above (L1), lateral to (L2), or absent from the region. These simulated CV values agree with the experimental porcine range of 27.9–43.6 cm/s measured in the MI region by OM under sinus and paced rhythms [1].

  The CV in the EHT was influenced by both its intrinsic conductivity and the underlying myocardium, suggesting that the host tissue effectively drove EHT depolarization, as reported in [10, 12, 21]. At the L1 position with low conductivity (C10), the EHT exhibited a CV of 13.6 cm/s, consistent with reported *in vitro* EHT values (5–24 cm/s) [15, 14]. Because the EHT at this location predominantly overlies non-conducting SZ or slow-conducting BZ, the simulated CVs naturally approximated *in vitro* values. In contrast, when assigned adult-like conductivity (C90), the CV in the EHT increased, particularly at the L2 position where it overlies fast-conducting HZ, approaching healthy myocardial velocities.

  Overall, this analysis confirms that the simulated CVs accurately reproduce porcine-specific experimental values under healthy and MI conditions, in both sinus and paced rhythms, as well as *in vitro* EHT dynamics, despite the non-uniform, bimodal mesh resolution. These results provide a solid foundation for our arrhythmia inducibility assessment. This quantitative accuracy is further supported by qualitative validation, in which simulated arrhythmic patterns were compared with subject-specific, OM-derived arrhythmic events (G1 simulations). Importantly, no additional adjustments to conductivity parameters were made using these OM data under arrhythmia, ensuring a clear separation between model calibration (based on OM of sinus and paced rhythms) and evaluation (based on OM of reentrant activity)."

- Mesh resolution-related uncertainties (Section 4.6): "The tetrahedralization process employed edge lengths consistent with established literature [2, 33, 6, 56], which have been shown to reliably capture both physiological and arrhythmic dynamics. Specifically, Boyle et al. [57] demonstrated that edge lengths below 400 µm are sufficient to resolve reentrant circuits consistent

with experimental observations while mitigating numerical conduction block artifacts. More recently, Bishop et al. [55] highlighted the need for finer discretizations ($\leq$ 200 µm) in low-CV regions to prevent spurious conduction block and reduce variability in arrhythmic outcomes. In our framework, median edge lengths below this 200 µm threshold were achieved within the EHT and adjacent regions. Furthermore, we applied the CV tuning strategies recommended by Bishop et al. [55] for coarser meshes: conductivities in infarcted regions were adjusted to match species-specific OM data obtained under sinus and paced rhythms [1], while settings in healthy and EHT regions produced CVs consistent with experimental benchmarks (see Section 2.6). Although coarser discretizations have been successfully used in prior studies [57, 56, 58, 10, 11, 2, 33], our multiscale meshing approach enhances the robustness of the inducibility assessment by combining high resolution in critical zones with validated conductivity selection."

Maturation and EHT electrophysiological alterations post-implantation: We agree with the Reviewer that maturation is a complex process involving multiple mechanisms that were not explicitly considered in this study. Accordingly, we have revised the title of Section 3.5 to "Contribution of EHT electrical coupling to arrhythmogenesis after MI", as suggested.

In our manuscript, we also acknowledge that: "hiPSC-CMs remain structurally and functionally immature compared to adult cardiomyocytes, even when cultured in biomimetic environments incorporating electromechanical stimulation and biochemical cues within bioprinted melt electrowriting scaffolds [15, 64]. Electrophysiologically, hiPSC-CMs exhibit spontaneous automaticity, elevated diastolic membrane potentials, prolonged action potentials (APs), and reduced upstroke velocities [65], resulting in suboptimal intercellular coupling and extended AP durations (APDs) in EHTs relative to the native myocardium [15, 64]". These features mechanistically underlie the fetal-like phenotype observed experimentally, which in the Paci et al. [23] model is primarily reflected by prolonged APD and cellular automaticity. At the tissue level, the reduced density of connexin-formed gap junctions is represented by lower cell-to-cell coupling within the EHT.

In our previous simulations [22, 12, 21], in which the EHT was coupled either to a slab of MI tissue or to a full BiV MI geometry under varying degrees of attachment and cellular alignment, we consistently found that once the EHT achieved at least minimal electrical coupling to the myocardium, its intrinsic automaticity was suppressed, and its depolarization and repolarization were driven by the underlying myocardium. Importantly, the degree of this dominance strongly depended on EHT conductivity. For example, in both slab configurations [1] and realistic BiV geometries [21], the APD within the EHT was modulated by the contacting tissue, remaining prolonged in regions adjacent to MI and shortened in regions adjacent to healthy myocardium.

For brevity, these observations have been concisely incorporated into the "Limitations, uncertainties, and future work" section, as they were analyzed in our prior publications. Additionally, under the subsection addressing "Extrinsic factors post-implantation of the EHT", we now discuss how these aspects may influence VT vulnerability and explicitly clarify that EHT automaticity was not intentionally suppressed (e.g., by blocking the inward hyperpolarization-activated funny current in the Paci et al. [23] model). Regarding EHT automaticity, we also state early in Section 2.4 that: "The intrinsic automaticity of the Paci et al. [23] model was preserved without modification. Stimulated prepacing was used to reflect standard preculturing protocols, in which external electrical stimulation is applied to enhance hiPSC-CM maturation [64]."

The revised text in Section 4.6 now reads:

"**Extrinsic factors post-implantation of the EHT:** External influences also affect remuscularization outcomes [14, 15, 16, 13, 10].

- Degree of attachment: Incomplete physical engraftment may result from air bubbles in poorly adhered constructs, scar tissue from stitching, immune-mediated EHT isolation in xenotransplantation, or shear stress during cardiac contraction-relaxation cycles [10, 15]. While our modeling of BiV-EHT coupling as a continuum interface provides a tractable framework for organ-scale VT assessment, representing the discrete, heterogeneous electrical coupling at the graft-host interface remains a key challenge. As noted by Gibbs et al. [17], spatiotemporal heterogeneities in interface coupling can create localized source-sink mismatches that strongly influence focal arrhythmia susceptibility. Prior simulations suggest that homogeneous partial engraftment increases VT risk, whereas heterogeneous partial versus complete engraftment shows comparable risk [10, 11]. Consistently, our previous computational study found only minor electrophysiolog-

ical variations across degrees of heterogeneous engraftment, provided minimal electrical contact existed [12]. Therefore, we focused on complete engraftment in this work.

- EHT-induced ectopy: Experimental studies report a high ectopic burden following cellular injection [16, 18, 13, 10]. Computational evidence indicates that ectopy arises under incomplete engraftment or extreme hiPSC-CM heterogeneity, but not under complete engraftment. Sustained VT further requires a bradycardic beat (or absence of other non-EHT pacing) [19, 17] and suppression of CS retrograde conduction [19]. Our results and others [10, 11, 20, 12, 21, 22] found no ectopic-driven VT, consistent with the high electrotonic load of the host myocardium suppressing spontaneous activity and the intrinsically slower EHT beating rate, ensuring pacing by the host myocardium. This effect persisted despite using the most recent Paci et al. [23] model, which features enhanced calcium handling and more robust spontaneous beating. Rapid host-induced pacing may further suppress hiPSC-CM automaticity by depleting sarcoplasmic reticulum calcium stores, aligning with findings by Riebel et al. [19]. Recent preclinical and clinical EHT studies [18, 24] reporting no arrhythmogenicity support these observations. However, confirmation of stable EHT-myocardium coupling and high cell survival remains necessary. Future studies could investigate bradycardic rhythms and heterogeneous grafts to model conditions more favorable to ectopy.

- Transitional phase: We, like others [10, 11], focused on baseline MI and post-EHT implantation endpoints. In this context, our previous work [22, 12, 21] showed that the host myocardium dominates both depolarization and repolarization of the EHT once minimal engraftment occurs. Specifically, APD values within the EHT mirrored adjacent myocardial regions. Intrinsic automaticity was suppressed even with the inward, hyperpolarization-activated funny current in the updated Paci et al. [23] model, and CV within the EHT followed that of neighboring myocardium, consistent with prior *in silico* reports [10, 11, 20]. Nevertheless, dynamic processes during graft integration, such as temporal changes in EHT-myocardium coupling and cell viability (not considered here), may substantially influence arrhythmogenicity. Liu et al. [13] reported early focal VT risk days post-injection, which transitions to relative stability over weeks. Similarly, computational studies show that focal arrhythmia susceptibility waxes and wanes as graft-host coupling gradually increases [17]. For reentrant mechanisms, spatiotemporal variations in electrical coupling may either facilitate or obstruct formation of low-conduction isthmuses (substrates for unidirectional block) and the sustainability of reentrant circuits, resulting in a highly variable VT burden during integration. Future simulations could incorporate time-varying conductivity, stochastic boundary coupling, and hiPSC-CM electrophysiology to capture this high-risk transitional phase between implantation and full electrical integration."

Subsection 4.2, how this protocol differs from the Arevalo et al. protocol? This protocol reminds me of an approach called PEERP. Could you please discuss this with your approach?

We thank the Reviewer for this comment. In the revised manuscript, Section 4.2 has been expanded to explicitly highlight the differences between our protocol and the methodology established by Arevalo et al. [2]. Furthermore, we have incorporated a discussion of the PEERP (Pacing at the End of the Effective Refractory Period) protocol proposed by Azzolin et al. [3]. We highlight evidence from their work demonstrating that a protocol with modest temporal granularity can successfully identify a broad range of arrhythmic activity, provided that premature stimuli are applied near the effective refractory period. In addition, we included the authors' observation of reproducing a great amount of the arrhythmic activity when using a lower number of pacing sites. The first part of the revised Section 4.2 now reads:

"Arrhythmia inducibility was assessed using an *in silico* AIP inspired by previous approaches, such as the Virtual Heart Arrhythmia Risk Predictor [2], but streamlined by employing fewer pacing sites, a shorter S1 train, and a single S2 stimulus delivered at four coupling intervals to balance physiological fidelity and computational tractability. Specifically, Arevalo et al. [2] applied eight S1 stimuli followed by three trains (S2-S3-S4) of premature extra-stimuli. In their study, each premature train was initially delivered 300 ms after the last S1 stimulus and shortened in 10 ms steps until loss of capture or arrhythmia induction. In contrast, we used four S1 stimuli followed by a single premature S2 stimulus (at 250, 265, 280, or 295 ms). The motivation for this reduction was that, under deterministic modeling, electrophysiological activity stabilizes after only a few beats, thereby

justifying a shorter pacing sequence. In line with this, Azzolin et al. [3] showed that as few as two stimuli can induce arrhythmia when delivered at the effective refractory period of the pacing site, and that additional premature stimuli rarely increased arrhythmia inducibility in computational atrial models. Accordingly, our protocol prioritizes identification of inducible substrates over exhaustive characterization of the full vulnerability window.

Furthermore, we adopted a more targeted distribution of pacing sites compared with the 19 locations (RV apex, RV outflow tract, and 17 AHA-based sites) evaluated by Arevalo et al. [2]. Our protocol used a subset of at least 5 pacing sites selected from those 19 locations. Because the BZ constitutes the critical substrate for reentry [4, 5, 6], pacing sites were distributed homogeneously around the MI border, as more distant pacing often elicits redundant reentrant patterns. Consistent with this, a comprehensive AIP applied to the atria showed that electrophysiologically remodeled regions exhibit greater arrhythmia susceptibility and that a reduced number of homogeneously distributed pacing sites can capture up to 90% of induced arrhythmias compared with more exhaustive protocols [3]. Overall, these strategic simplifications enabled high-throughput assessment of the CS-BiV complex while preserving substantial computational scale, yielding reentrant patterns that closely resemble experimental observations (see Section 3.1).

The selection of S2 coupling intervals was informed by both established literature [2, 7, 6, 8] and the specific electrophysiological constraints of our porcine BiV models. While Arevalo et al. [2] utilized a sequence of premature stimuli, initially delivered 300 ms after the last S1 stimulus with 10 ms decrements, other studies adapting this method to specific porcine or human phenotypes have employed S2 values starting at 250 ms [7, 6, 8]. In our study, due to HZ/BZ refractoriness, as defined by the Gaur et al. [9] cellular model and its experimentally calibrated tissue properties [1], short S2 intervals or near-MI pacing often failed to capture, preventing VT induction. Longer S2 delays increased inducibility, and variability in such inducibility across S2 values highlighted the value of selecting the shortest effective S2 and identifying the S2 delay that produces the highest inducibility for post-MI remuscularization analyses (G3 and G4 simulations). This establishes a robust framework for assessing inducibility with the Gaur et al. [9] model embedded in BiV simulations."

Furthermore, we extended Section 4.6 on "Limitations, uncertainties, and future work" to broadly discuss possible improvements for further standardizing the AIP with techniques described in [3]:

"***In silico* AIP:** The combination of a limited, low-resolution S2 range and a reduced number of pacing sites, rather than a high-resolution exploration of coupling intervals or a complete 19-site sweep, may fail to capture narrow spatiotemporal windows of vulnerability for VT induction. Consequently, models classified as non-inducible under our protocol could potentially exhibit reentry under a more exhaustive coupling-interval search. Thus, although our *in silico* AIP qualitatively reproduced subject-specific experimental VT patterns with substantially lower computational cost, future studies may benefit from more spatially detailed and temporally extended stimulation strategies. From a spatial standpoint, although no fully automated quantitative criteria were used for pacing-site selection, we implemented a reproducible strategy designed to homogeneously encircle the MI region and assess proarrhythmic BZ heterogeneity [5, 4, 3]. Future work could incorporate automated methods based on geodesic distance to scar tissue or the identification of slow-conducting isthmuses, as proposed by Campos et al. [8]. Temporally, broader and site-specific S2 coupling interval ranges could be examined using checkpointing strategies and automated estimation of the effective refractory period at each pacing site, following approaches similar to those described by Azzolin et al. [3]. Collectively, these methodological refinements would further standardize the AIP and enable systematic identification of the key spatial and temporal pacing features underlying arrhythmia induction, while maintaining computationally feasibility. Such advances may uncover reentry mechanisms that arise only within narrow temporal windows or specific trigger-substrate configurations."

Line 471, how physiological is your CS? Could you please compare this to other simulation studies that have shown this capacitive effect?

We thank the Reviewer for the opportunity to clarify the physiological fidelity of our CS representation and to better contextualize the capacitive-like effect observed in our simulations by comparison with prior computational studies.

Physiological realism of the CS: The CS architecture employed in this study was defined and

extensively validated in our previous work [1], in which multiple CS configurations were systematically evaluated and compared against porcine experimental data at both tissue and organ scales. In this answer to the Reviewer, we briefly summarize the construction criteria and validation outcomes most relevant to the present work.

The His bundle was generated by connecting anatomically defined landmarks using one-dimensional elements, following [47]. The atrioventricular node was positioned 5 mm above the septal base toward the anterior RV. The His bundle extended toward the apex and bifurcated into left and right branches, with the left branch further subdividing into anterior and posterior fascicles. Consistent with experimental observations, the LV bundle branches terminated at papillary muscles identified from cine-CMR [66, 67], while the RV branch terminated more basally along the septomarginal trabecula, in agreement with porcine anatomy [46].

The Purkinje network was generated from the bundle branch endpoints using a fractal tree algorithm [48], resulting in an initial endocardial CS. Given experimental evidence that the porcine CS lies deeper within the ventricular wall than in humans [46], an alternative intramyocardial CS was constructed by projecting CS fibers into the subendocardium and, for 75% of branches, into the mid-myocardium [61]. PMJs were distributed throughout both ventricles, excluding regions known to exhibit delayed activation [30].

To further explore CS architecture, four CS variants were constructed (see [1]) and compared against experimental ECG and OM data in both healthy and MI conditions. Among these, one configuration involving an intramyocardial location, PMJs located at the septal region of the RV and a more apical position for the endpoints of the left bundle branches provided the best agreement with porcine experimental observations. Specifically, *in silico* maximum activation times and depolarization patterns were in good agreement with porcine-specific OM data under both healthy and MI conditions. Regarding depolarization patterns, the first epicardial breakthrough occurred in the RV area pretrabecularis, and epicardial activation predominantly propagated from the RV to the LV in both anterior and posterior views, consistent with porcine OM-derived experimental observations. Moreover, comparison with healthy ECG recordings showed that this CS architecture yielded a mean Pearson correlation of 0.75 across all leads and healthy samples between simulated and experimental QRS complexes. These validations, together with consistency with large-mammal literature [30, 46], support the physiological relevance of the CS used in the present study.

To clearly direct the reader to our previous work and to provide additional relevant details in the present study, we have expanded or added the following sections in the revised manuscript:

- Section 2.1: "The CS was generated for each model using a fractal tree algorithm and subsequently projected into the ventricular wall, following established methods [47, 48]. The CS mesh was defined using line elements with a mean length of 528 µm, incorporating an average of 1623 Purkinje-muscular junctions (PMJs). CS-activated ventricular nodes were located within a maximum distance of 0.5 mm from the CS endpoints, with an average of 14.5 connections between each endpoint and the myocardial nodes to avoid source-sink mismatch-mediated conduction blocks at PMJs [1]. Detailed descriptions of the BiV MI and CS model construction and their validation against porcine-specific experimental data are provided in our previous work [1]."

- Section 2.4: "Similarly, although the CS LDC was set to achieve CVs characteristic of the human CS ($\approx$ 200 cm/s) [30], it successfully reproduced the experimental, OM-derived maximum activation times and depolarization patterns of the anterior porcine heart under sinus rhythm [1]. The LDC for the CS was reduced near its endpoints using a sigmoid profile to match the ventricular LDC, as proposed by Dux-Santoy et al. [31], thereby enabling retrograde propagation at the PMJs. Bidirectional conduction across the PMJs was achieved (see S1 Figure). A minimal anterograde propagation delay of 1.1 ms was observed, whereas the retrograde delay was zero (see discussion in S1 Section and Section 4.6)."

- Supporting S1 Section: "As shown in S1 Figure, both anterograde and retrograde electrical propagation across the PMJs was successfully captured. To quantify direction-dependent propagation delays, a localized region of the CS-BiV complex near pacing site 15 (pig 6) was analyzed following the final S1 stimulus. The propagation delay at a PMJ was defined as the mean activation time difference between the CS endpoint and its associated myocardial nodes. As defined in Section 2.1, these myocardial nodes were identified as ventricular nodes within a 0.5 mm radius

of the CS endpoint. Across the full CS-BiV domain, complex phenomena can introduce confounding effects that preclude a clean calculation. These include the simultaneous depolarization of a PMJ from both the CS and myocardial tissue, as well as variations in local PMJ density that alter the source–sink mismatch and modify conduction delays by changing electrotonic loading effects [60]. Consequently, the analysis was restricted to a reduced postero-apical region near the pacing site, focusing specifically on the three PMJ propagation events highlighted in S1 Figure.

As illustrated in S1 Figure, three epicardial breakthroughs are observed: one (red star in S1 Figure) originating from stimulation at endocardial pacing site 15, producing a retrograde reentry, and two (black stars in S1 Figure) resulting from anterograde depolarization through distinct PMJs. The retrograde delay was zero, suggesting that the activation of the CS endpoint occurred simultaneously with that of the connected myocardial nodes. However, this measurement was limited by the temporal resolution of our simulations (1 ms). For anterograde conduction, a mean delay of 1.1 ms was measured across the two PMJs propagating in the anterograde direction.

Computational studies of bidirectional conduction have calibrated higher delay values, particularly in human-oriented modeling and simulation, largely based on canine experimental data. For example, Gillette et al. [49] used anterograde and retrograde propagation delays of 8 and 3 ms, respectively. In closer agreement with the present work, Riebel et al. [19] used delays of 2 and 1 ms for anterograde and retrograde propagation. Experimentally, Veenstra et al. [62] reported anterograde delays of 2–4 ms in canine studies and reviewed evidence suggesting that anterograde delays below 5 ms are more physiological than larger values. Retrograde delays are consistently reported to be shorter than anterograde delays, reflecting faster myocardium-to-CS conduction, likely due to the greater electrotonic load imposed on the CS endpoint by the bulkier myocardial tissue, a source–sink mismatch widely observed *in silico* [63, 59, 1]. We previously showed that this effect is accentuated in pigs [1], as PMJs are located deeper within the myocardium than in dogs and humans and contact a larger myocardial mass for a given PMJ size [46, 61], thereby supporting the lower retrograde delay values obtained in the present study. Under this hypothesis, larger anterograde delays than those reported experimentally in dogs would also be expected. Indeed, when myocardial nodes located farther from the CS endpoint are considered in the anterograde delay calculation, higher values are obtained (mean anterograde delays of 1.7 and 2.7 ms for myocardial nodes located up to 0.6 and 1 mm from the CS endpoint, respectively). This observation is particularly relevant given that the experimental measurements used were inherently limited in spatial resolution and are typically unable to resolve distances smaller than 1 mm [62].

Taken together, we contend that the anterograde and retrograde delays employed here are consistent with physiological values: anterograde delays are systematically greater than retrograde delays, and the retrograde delay can reasonably be assumed to be zero due to the increased source–sink mismatch associated with the deeper CS in pigs. Future studies may further refine these parameters by incorporating porcine-specific experimental data, which are currently lacking."

- Section 4.3: "Using anatomically realistic CS distributions (details on construction and validation are provided in [1]), ..."

On the capacitive-like effect: In our work, the term "capacitive effect" was introduced to describe a phenomenon in which a slowly depolarized MI region, characterized by long APD and reduced CV, maintains a high V_m long enough to reexcite surrounding tissue once excitability is recovered, thereby sustaining the VT. While this terminology is not commonly used in the literature, the underlying mechanism has been observed in prior *in silico* studies under similar electrophysiological conditions.

For instance, Riebel et al. [19] simulated a human BiV MI model with retrograde CS conduction and hiPSC-CM grafts. In that study, a CS-generated activation slowly depolarized the MI region near the end of its refractory period. Once adjacent endocardial tissue regained excitability, the MI region acted as a delayed source, releasing depolarization into healthy myocardium and propagating retrogradely into the CS.

To clarify this point and following the Reviewer's comment, we have revised the manuscript text to explicitly state that, although not labeled as capacitive in prior studies, this behavior can arise when-

ever regions with prolonged APD and slow conduction are depolarized in the presence of heterogeneous recovery dynamics. The revised Section 4.3 now reads:

"These electrical shortcuts may sustain or terminate reentry or alter MI depolarization timing, facilitating or impairing the phenomenon we term the *capacitive effect*. Although not explicitly labeled as such in other studies, this effect occurs when a region with prolonged APD and slow conductivity (such as the MI or EHT-treated zone) remains activated until the surrounding healthy tissue with a shorter APD recovers excitability. A comparable mechanism was observed by Riebel et al. [19], in which a CS-originated activation slowly traversed the MI, subsequently reexciting the healthy endocardium and propagating retrogradely back into the CS after the refractory period of the HZ had ended."

Lines 550-553, how was the conduction velocity to the optical mapping experiments? If I understood it correctly the experiments were under VT, how did the authors calibrate the model using this information? Please add an explicit section on the calibration of conduction velocity from the optical mapping experiments under VT.

We thank the Reviewer for highlighting the need to clarify how OM data were used for CV calibration and validation.

As noted in our previous responses, the calibration of the electrophysiological model was not performed within the present study but was carried out in our prior work [1], in which extensive porcine-specific experimental data were available. In that study, tissue-level OM data and organ-level electrocardiogram recordings were used under both healthy and MI conditions. Specifically, OM data acquired during sinus rhythm or controlled pacing were employed to validate *in silico* maximum activation times, CVs, and depolarization patterns in the healthy case, and to calibrate CV and action potential duration in the MI border zone. Importantly, OM data acquired during VT were not used for calibration.

In the present work, OM recordings obtained during VT were used exclusively for the validation of simulated reentrant patterns. As a result, reentrant activity in the experimental data is faster than in the *in silico* simulations. While an additional calibration step using OM data acquired during VT could be performed, our objective here was not to generate an exact quantitative replica of the experimental VT, but rather to assess whether the model could reproduce the main qualitative and mechanistic features of VT dynamics observed experimentally.

To explicitly clarify this workflow, we have added a new Section 2.6 describing the calibration and validation of CV using OM data, including a clear distinction between the use of OM under sinus or paced rhythms for validation or calibration, and of OM under VT for validation. In addition, we have expanded the discussion in the "Limitations, uncertainties, and future work" section to address the concerns of mesh discretization on effective CV representation, especially on bimodal edge-length meshes incorporating the EHT. These Sections now read:

- Section 2.6: "In Section 2.4, conductivity values for the CS and HZ were defined based on existing large-mammal literature (canine [34] and human [29]) and validated against our own porcine-specific experimental data (electrocardiogram and OM) obtained under sinus or paced rhythm (see our previous work [1]). Additionally, BZ conductivities were adjusted to reproduce post-MI conduction CVs previously measured via OM under sinus or paced rhythm [1]. In the present work, inclusion of the EHT modified the computational domain, requiring mesh refinement in the remuscularized region (see Section 2.3). To ensure that the selected conductivities, together with the non-uniform mesh resolution, preserved physiological accuracy, we evaluated the simulated CVs.

  CVs were calculated across the epicardial faces of the BiV meshes to maintain consistency with the superficial nature of OM data [1]. The CV magnitude between two nodes was computed as the ratio of their Euclidean distance to the difference in their activation times. For each node, a mean CV vector was calculated within a defined radius [1]. As shown in S2 Figure, multiple radii were evaluated; smaller radii (e.g., 0.6 mm) produced numerical artifacts, specifically infinite CVs (red in S2 Figure), due to the 1 ms activation time resolution and the proximity of nodes with identical activation times, particularly in fast-conducting HZ. Conversely, larger radii yielded more biomimetic CVs in the HZ, with low variation upon further radial increments. Accordingly,

a 2 mm radius was selected as the minimum threshold providing consistent, finite CV values in the HZ.

Under this configuration, CVs were computed for the final S1 stimulus in pig 6 (pacing site 15) across all modeling scenarios: the baseline MI model without EHT, and models with the EHT positioned at L1 and L2 under C10 and C90 conductivity settings (Fig 4). In the HZ, simulated CVs ranged from 69–79 cm/s. Despite differences in mesh resolution among cases, HZ values remained consistent and, notably, closely matched the experimental healthy porcine CV of 74.2 cm/s, previously measured by OM under sinus and paced rhythm [1]. Lower CV values were primarily attributable to the presence of the slower-conducting EHT (notably the L2-C10 configuration) rather than to variations in mesh edge length. Indeed, as shown in Fig 4a, CVs within the finely meshed EHT region were comparable to those of the underlying myocardium (BZ or HZ) and were consistent with the values obtained in more distant, coarser-meshed equivalent (BZ or HZ) regions. Similarly, CVs in the BZ were robust to changes in mesh resolution. Values were nearly identical (27 cm/s for C10; 34 cm/s for baseline MI and C90) regardless of whether the EHT was implanted directly above (L1), lateral to (L2), or absent from the region. These simulated CV values agree with the experimental porcine range of 27.9–43.6 cm/s measured in the MI region by OM under sinus and paced rhythms [1].

The CV in the EHT was influenced by both its intrinsic conductivity and the underlying myocardium, suggesting that the host tissue effectively drove EHT depolarization, as reported in [10, 12, 21]. At the L1 position with low conductivity (C10), the EHT exhibited a CV of 13.6 cm/s, consistent with reported *in vitro* EHT values (5–24 cm/s) [15, 14]. Because the EHT at this location predominantly overlies non-conducting SZ or slow-conducting BZ, the simulated CVs naturally approximated *in vitro* values. In contrast, when assigned adult-like conductivity (C90), the CV in the EHT increased, particularly at the L2 position where it overlies fast-conducting HZ, approaching healthy myocardial velocities.

Overall, this analysis confirms that the simulated CVs accurately reproduce porcine-specific experimental values under healthy and MI conditions, in both sinus and paced rhythms, as well as *in vitro* EHT dynamics, despite the non-uniform, bimodal mesh resolution. These results provide a solid foundation for our arrhythmia inducibility assessment. This quantitative accuracy is further supported by qualitative validation, in which simulated arrhythmic patterns were compared with subject-specific, OM-derived arrhythmic events (G1 simulations). Importantly, no additional adjustments to conductivity parameters were made using these OM data under arrhythmia, ensuring a clear separation between model calibration (based on OM of sinus and paced rhythms) and evaluation (based on OM of reentrant activity)."

- Section 4.6: "The tetrahedralization process employed edge lengths consistent with established literature [2, 33, 6, 56], which have been shown to reliably capture both physiological and arrhythmic dynamics. Specifically, Boyle et al. [57] demonstrated that edge lengths below 400 µm are sufficient to resolve reentrant circuits consistent with experimental observations while mitigating numerical conduction block artifacts. More recently, Bishop et al. [55] highlighted the need for finer discretizations ($\leq$ 200 µm) in low-CV regions to prevent spurious conduction block and reduce variability in arrhythmic outcomes. In our framework, median edge lengths below this 200 µm threshold were achieved within the EHT and adjacent regions. Furthermore, we applied the CV tuning strategies recommended by Bishop et al. [55] for coarser meshes: conductivities in infarcted regions were adjusted to match species-specific OM data obtained under sinus and paced rhythms [1], while settings in healthy and EHT regions produced CVs consistent with experimental benchmarks (see Section 2.6). Although coarser discretizations have been successfully used in prior studies [57, 56, 58, 10, 11, 2, 33], our multiscale meshing approach enhances the robustness of the inducibility assessment by combining high resolution in critical zones with validated conductivity selection."

Finally, in our previous work [1], all experimental data, simulation results, and source code were made publicly available to ensure reproducibility. Moreover, the OM data acquired during VT and used in the present study are also available in the shared repository referenced in the "Data availability" section, enabling future studies to further calibrate tissue-level CV using OM data not only from sinus or paced rhythms but also during VT.

Minor comments:

Line 13, fibrotic tissue can contract. Fibrotic tissue does not have an "active" contraction as myocytes but it has "passive" properties which are stiffer than the healthy myocardium but does not mean that fibrotic tissue can not mechanically be deformed. I suggest rephrasing "with non-contractile fibrotic tissue".

We thank the Reviewer for this important clarification. We agree that fibrotic tissue does not exhibit active contraction like healthy myocardium but does retain passive mechanical properties and can be mechanically deformed, albeit with increased stiffness.

Accordingly, we have rephrased the text to avoid the implication that fibrotic tissue is entirely non-contractile. The text has been rephrased to "This loss of cardiomyocytes initiates a coordinated inflammatory response that ultimately replaces the necrotic myocardium with fibrotic tissue devoid of active contractile properties, leading to impaired systolic and diastolic function [68]."

Line 149, I suggest changing the word discretize to tetrahedralized as the surface mesh is already discretized.

Following the Reviewer's comment, we have modified "discretized" for "tetrahedralized" in Section 2.3

Line 170, conductance should be with capital g.

We thank the Reviewer for pointing this out. We have changed all the conductances throughout the text to capital "G".

References

[1] Rosales RM, Wu M, Claus P, Janssens S, Ríos-Muñoz GR, Fernández-Santos ME, et al. Integrated multi-modal data analysis for computational modeling of healthy and location-dependent myocardial infarction conditions in porcine hearts [Preprint]. bioRxiv; 2025 [cited 2025 November 5]. Available from: https://doi.org/10.1101/2025.10.31.685788.

[2] Arevalo HJ, Vadakkumpadan F, Guallar E, Jebb A, Malamas P, Wu KC, et al. Arrhythmia risk stratification of patients after myocardial infarction using personalized heart models. Nature Communications. 2016;7:11437. doi:10.1038/ncomms11437.

[3] Azzolin L, Schuler S, Dössel O, Loewe A. A reproducible protocol to assess arrhythmia vulnerability in silico: Pacing at the end of the effective refractory period. Frontiers in Physiology. 2021;12:656411. doi:10.3389/fphys.2021.656411.

[4] Schmidt A, Azevedo CF, Cheng A, Gupta SN, Bluemke DA, Foo TK, et al. Infarct tissue heterogeneity by magnetic resonance imaging identifies enhanced cardiac arrhythmia susceptibility in patients with left ventricular dysfunction. Circulation. 2007;115:2006-14. doi:10.1161/CIRCULATIONAHA.106.653568.

[5] Arevalo H, Plank G, Helm P, Halperin H, Trayanova N. Tachycardia in post-infarction hearts: Insights from 3D image-based ventricular models. PLoS ONE. 2013;8:e68872. doi:10.1371/journal.pone.0068872.

[6] Mendonca Costa C, Gemmell P, Elliott MK, Whitaker J, Campos FO, Strocchi M, et al. Determining anatomical and electrophysiological detail requirements for computational ventricular models of porcine myocardial infarction. Computers in Biology and Medicine. 2022;141:105061. doi:10.1016/j.compbiomed.2021.105061.

[7] Prakosa A, Arevalo HJ, Deng D, Boyle PM, Nikolov PP, Ashikaga H, et al. Personalized virtual-heart technology for guiding the ablation of infarct-related ventricular tachycardia. Nature Biomedical Engineering. 2018;2:732-40. doi:10.1038/s41551-018-0282-2.

[8] Campos FO, Neic A, Mendonca Costa C, Whitaker J, O'Neill M, Razavi R, et al. An automated near-real time computational method for induction and treatment of scar-related ventricular tachycardias. Medical Image Analysis. 2022;80:102483. doi:10.1016/j.media.2022.102483.

[9] Gaur N, Qi XY, Benoist D, Bernus O, Coronel R, Nattel S, et al. A computational model of pig ventricular cardiomyocyte electrophysiology and calcium handling: Translation from pig to human electrophysiology. PLoS Computational Biology. 2021;17:e1009137. doi:10.1371/journal.pcbi.1009137.

[10] Yu JK, Liang JA, Franceschi WH, Huang Q, Pashakhanloo F, Sung E, et al. Assessment of arrhythmia mechanism and burden of the infarcted ventricles following remuscularization with pluripotent stem cell-derived cardiomyocyte patches using patient-derived models. Cardiovascular Research. 2022;118:1247-61. doi:10.1093/cvr/cvab140.

[11] Fassina D, M Costa C, Bishop M, Plank G, Whitaker J, Harding SE, et al. Assessing the arrhythmogenic risk of engineered heart tissue patches through *in silico* application on infarcted ventricle models. Computers in Biology and Medicine. 2023;154:106550. doi:10.1016/j.compbiomed.2023.106550.

[12] Rosales RM, Mountris K, Doblaré M, Mazo M, Pueyo E. *In silico* assessment of arrhythmic risk in infarcted ventricles engrafted with engineered heart tissues. In: 2023 Computing in Cardiology (CinC); 2023. p. 1-4. doi:10.22489/CinC.2023.275.

[13] Liu YW, Chen B, Yang X, Fugate JA, Kalucki FA, Futakuchi-Tsuchida A, et al. Human embryonic stem cell–derived cardiomyocytes restore function in infarcted hearts of non-human primates. Nature Biotechnology. 2018;36:597-605. doi:10.1038/nbt.4162.

[14] Montero-Calle P, Flandes-Iparraguirre M, Mountris K, S de la Nava A, Laita N, Rosales RM, et al. Fabrication of human myocardium using multidimensional modelling of engineered tissues. Biofabrication. 2022;14:045017. doi:10.1088/1758-5090/ac8cb3.

[15] Iglesias-García O, Flandes-Iparraguirre M, Montero-Calle P, Rosales RM, Ullate-Agote A, Sánchez-Bueno A, et al. Biologically-inspired melt electrowriting for the generation of highly biomimetic functional myocardium. Advanced Functional Materials. 2025;35:2420106. doi:10.1002/adfm.202420106.

[16] Marchiano S, Nakamura K, Reinecke H, Neidig L, Lai M, Kadota S, et al. Gene editing to prevent ventricular arrhythmias associated with cardiomyocyte cell therapy. Cell Stem Cell. 2023;30:396-414.e9. doi:10.1016/j.stem.2023.04.010.

[17] Gibbs CE, Marchianó S, Zhang K, Yang X, Murry CE, Boyle PM. Graft-host coupling changes can lead to engraftment arrhythmia: A computational study. The Journal of Physiology. 2023;601:2733-49. doi:10.1113/JP284244.

[18] Jebran AF, Seidler T, Tiburcy M, Daskalaki M, Kutschka I, Fujita B, et al. Engineered heart muscle allografts for heart repair in primates and humans. Nature. 2025;639:503-11. doi:10.1038/s41586-024-08463-0.

[19] Riebel LL, Wang ZJ, Martinez-Navarro H, Trovato C, Camps J, Berg LA, et al. *In silico* evaluation of cell therapy in acute versus chronic infarction: Role of automaticity, heterogeneity and Purkinje in human. Scientific Reports. 2024;14:21584. doi:10.1038/s41598-024-67951-5.

[20] Fassina D, Costa CM, Longobardi S, Karabelas E, Plank G, Harding SE, et al. Modelling the interaction between stem cells derived cardiomyocytes patches and host myocardium to aid non-arrhythmic engineered heart tissue design. PLoS Computational Biology. 2022;18:e1010030. doi:10.1371/journal.pcbi.1010030.

[21] Rosales RM, Doblaré M, M Mazo Vega M, Pueyo E. Design of engineered heart tissues to minimize arrhythmic risk after implantation in infarcted hearts. In: 2024 Computing in Cardiology (CinC); 2024. p. 1-4. doi:10.22489/CinC.2024.376.

[22] Rosales RM, Mountris KA, Oliván-Viguera A, Pérez-Zabalza M, Cedillo-Servin G, Iglesias-García O, et al. Experimentally-guided *in silico* design of engineered heart tissues to improve cardiac electrical function after myocardial infarction. Computers in Biology and Medicine. 2024;171:108044. doi:10.1016/j.compbiomed.2024.108044.

[23] Paci M, Passini E, Klimas A, Severi S, Hyttinen J, Rodriguez B, et al. All-optical electrophysiology refines populations of *in silico* human iPSC-CMs for drug evaluation. Biophysical Journal. 2020;118:2596-611. doi:10.1016/j.bpj.2020.03.018.

[24] Ensminger S, Kutschka I, Brandenburg S, Bremmer F, Didie M, Eitel I, et al. Two-center surgical experience from the first-in-human BioVAT-HF-DZHK20 clinical trial. The Journal of Heart and Lung Transplantation. 2025;44:S64-5. doi:10.1016/j.healun.2025.02.136.

[25] Drouin E, Charpentier F, Gauthier C, Laurent K, Le Marec H. Electrophysiologic characteristics of cells spanning the left ventricular wall of human heart: Evidence for presence of M cells. Journal of the American College of Cardiology. 1995;26:185-92. doi:10.1016/0735-1097(95)00167-X.

[26] Pueyo E, Corrias A, Virág L, Jost N, Szél T, Varró A, et al. A multiscale investigation of repolarization variability and its role in cardiac arrhythmogenesis. Biophysical Journal. 2011;101:2892-902. doi:10.1016/j.bpj.2011.09.060.

[27] Perotti LE, Krishnamoorthi S, Borgstrom NP, Ennis DB, Klug WS. Regional segmentation of ventricular models to achieve repolarization dispersion in cardiac electrophysiology modeling. International Journal for Numerical Methods in Biomedical Engineering. 2015;31:e02718. doi:10.1002/cnm.2718.

[28] Stewart P, Aslanidi OV, Noble D, Noble PJ, Boyett MR, Zhang H. Mathematical models of the electrical action potential of Purkinje fibre cells. Philosophical Transactions of the Royal Society A: Mathematical, Physical and Engineering Sciences. 2009;367:2225-55. doi:10.1098/rsta.2008.0283.

[29] Potse M, Dubé B, Richer J, Vinet A, Gulrajani RM. A comparison of monodomain and bidomain reaction-diffusion models for action potential propagation in the human heart. IEEE Transactions on Biomedical Engineering. 2006;53:11. doi:10.1109/TBME.2006.880875.

[30] Durrer D, Van Dam RT, Freud GE, Janse MJ, Meijler FL, Arzbaecher RC. Total excitation of the isolated human heart. Circulation. 1970;41:899-912. doi:10.1161/01.CIR.41.6.899.

[31] Dux-Santoy L, Sebastian R, Rodriguez JF, Ferrero JM. Modeling the different sections of the cardiac conduction system to obtain realistic electrocardiograms. In: 2013 35th Annual International Conference of the IEEE Engineering in Medicine and Biology Society (EMBC); 2013. p. 6846-9. doi:10.1109/EMBC.2013.6611130.

[32] Pu J, Boyden PA. Alterations of Na^+ currents in myocytes from epicardial border zone of the infarcted heart. Circulation Research. 1997;81:110-9. doi:10.1161/01.RES.81.1.110.

[33] Lopez-Perez A, Sebastian R, Izquierdo M, Ruiz R, Bishop M, Ferrero JM. Personalized cardiac computational models: From clinical data to simulation of infarct-related ventricular tachycardia. Frontiers in Physiology. 2019;10:580. doi:10.3389/fphys.2019.00580.

[34] Spach MS, Miller WT, Geselowitz DB, Barr RC, Kootsey JM, Johnson EA. The discontinuous nature of propagation in normal canine cardiac muscle. Evidence for recurrent discontinuities of intracellular resistance that affect the membrane currents. Circulation Research. 1981;48:39-54. doi:10.1161/01.RES.48.1.39.

[35] Campos FO, Whitaker J, Neji R, Roujol S, O'Neill M, Plank G, et al. Factors promoting conduction slowing as substrates for block and reentry in infarcted hearts. Biophysical Journal. 2019;117:2361-74. doi:10.1016/j.bpj.2019.08.008.

[36] Olvera D, Sohrabi Molina M, Hendy G, Monaghan MG. Electroconductive melt electrowritten patches matching the mechanical anisotropy of human myocardium. Advanced Functional Materials. 2020;30:1909880. doi:10.1002/adfm.201909880.

[37] Roshanbinfar K, Kolesnik-Gray M, Angeloni M, Schruefer S, Fiedler M, Schubert DW, et al. Collagen hydrogel containing polyethylenimine-gold nanoparticles for drug release and enhanced beating properties of engineered cardiac tissues. Advanced Healthcare Materials. 2023;12:2202408. doi:10.1002/adhm.202202408.

[38] Boyle PM, Massé S, Nanthakumar K, Vigmond EJ. Transmural IK(ATP) heterogeneity as a determinant of activation rate gradient during early ventricular fibrillation: Mechanistic insights from rabbit ventricular models. Heart Rhythm. 2013;10:1710-7. doi:https://doi.org/10.1016/j.hrthm.2013.08.010.

[39] Lou X, Tang Y, Ye L, Pretorius D, Fast VG, Kahn-Krell AM, et al. Cardiac muscle patches containing four types of cardiac cells derived from human pluripotent stem cells improve recovery from cardiac injury in mice. Cardiovascular Research. 2023;119:1062-76. doi:10.1093/cvr/cvad004.

[40] Gao L, Gregorich ZR, Zhu W, Mattapally S, Oduk Y, Lou X, et al. Large cardiac muscle patches engineered from human induced-pluripotent stem cell–derived cardiac cells improve recovery from myocardial infarction in swine. Circulation. 2018;137:1712-30. doi:10.1161/CIRCULATIONAHA.117.030785.

[41] Ye L, Chang YH, Xiong Q, Zhang P, Zhang L, Somasundaram P, et al. Cardiac repair in a porcine model of acute myocardial infarction with human induced pluripotent stem cell-derived cardiovascular cells. Cell Stem Cell. 2014;15:750-61. doi:10.1016/j.stem.2014.11.009.

[42] Paci M, Koivumäki JT, Lu HR, Gallacher DJ, Passini E, Rodriguez B. Comparison of the simulated response of three *in silico* human stem cell-derived cardiomyocytes models and *in vitro* data under 15 drug actions. Frontiers in Pharmacology. 2021;12. doi:10.3389/fphar.2021.604713.

[43] Bhagirath P, Strocchi M, Bishop MJ, Boyle PM, Plank G. From bits to bedside: entering the age of digital twins in cardiac electrophysiology. Europace. 2024;26:euae295. doi:10.1093/europace/euae295.

[44] Camara O, Sermesant M, Lamata P, Wang L, Pop M, Relan J, et al. Inter-model consistency and complementarity: Learning from ex-vivo imaging and electrophysiological data towards an integrated understanding of cardiac physiology. Progress in Biophysics and Molecular Biology. 2011;107:122-33. doi:10.1016/j.pbiomolbio.2011.07.007.

[45] Albors C, Lluch È, Gomez JF, Cedilnik N, Mountris KA, Mansi T, et al. Meshless electrophysiological modeling of cardiac resynchronization therapy—benchmark analysis with finite-element methods in experimental data. Applied Sciences. 2022;12:6438. doi:10.3390/app12136438.

[46] Lelovas PP, Kostomitsopoulos NG, Xanthos TT. A comparative anatomic and physiologic overview of the porcine heart. Journal of the American Association for Laboratory Animal Science. 2014;53:432-8. Available from: `https://pmc.ncbi.nlm.nih.gov/articles/PMC4181683/`.

[47] Rosales RM, Mountris KA, Doblaré M, Mazo MM, Pueyo E. Ventricular conduction system modeling for electrophysiological simulation of the porcine heart. In: 2022 Computing in Cardiology (CinC); 2022. p. 1-4. doi:10.22489/CinC.2022.030.

[48] Sahli Costabal F, Hurtado DE, Kuhl E. Generating Purkinje networks in the human heart. Journal of Biomechanics. 2016;49:2455-65. doi:10.1016/j.jbiomech.2015.12.025.

[49] Gillette K, Gsell MAF, Bouyssier J, Prassl AJ, Neic A, Vigmond EJ, et al. Automated framework for the inclusion of a His–Purkinje system in cardiac digital twins of ventricular electrophysiology. Annals of Biomedical Engineering. 2021;49:3143-53. doi:10.1007/s10439-021-02825-9.

[50] Camps J, Berg LA, Wang ZJ, Sebastian R, Riebel LL, Doste R, et al. Digital twinning of the human ventricular activation sequence to clinical 12-lead ECGs and magnetic resonance imaging using realistic Purkinje networks for *in silico* clinical trials. Medical Image Analysis. 2024;94:103108. doi:10.1016/j.media.2024.103108.

[51] Sayers JR, Martinez-Navarro H, Sun X, De Villiers C, Sigal S, Weinberger M, et al. Cardiac conduction system regeneration prevents arrhythmias after myocardial infarction. Nature Cardiovascular Research. 2025;4:163-79. doi:10.1038/s44161-024-00586-x.

[52] Zhou Q, Grinspun E, Zorin D, Jacobson A. Mesh arrangements for solid geometry. ACM Transactions on Graphics. 2016;35:1-15. doi:10.1145/2897824.2925901.

[53] Cignoni P, Callieri M, Corsini M, Dellepiane M, Ganovelli F, Ranzuglia G. Meshlab: An open-source mesh processing tool. In: Eurographics Italian Chapter Conference; 2008. doi:10.2312/LocalChapterEvents/ItalChap/ItalianChapConf2008/129-136.

[54] Hoppe H, DeRose T, Duchamp T, McDonald J, Stuetzle W. Mesh optimization. In: Proceedings of the 20th Annual Conference on Computer Graphics and Interactive Techniques; 1993. p. 19–26. doi:10.1145/166117.166119.

[55] Bishop MJ, Plank G. Stochastic virtual heart model predictions. Nature Cardiovascular Research. 2025;4:539-42. doi:10.1038/s44161-025-00641-1.

[56] Sung E, Prakosa A, Zhou S, Berger RD, Chrispin J, Nazarian S, et al. Fat infiltration in the infarcted heart as a paradigm for ventricular arrhythmias. Nature Cardiovascular Research. 2022;1:933-45. doi:10.1038/s44161-022-00133-6.

[57] Boyle PM, Ochs AR, Ali RL, Paliwal N, Trayanova NA. Characterizing the arrhythmogenic substrate in personalized models of atrial fibrillation: Sensitivity to mesh resolution and pacing protocol in AF models. EP Europace. 2021;23:i3-i11. doi:10.1093/europace/euaa385.

[58] Sung E, Prakosa A, Trayanova N. Reply to: Stochastic virtual heart model predictions. Nature Cardiovascular Research. 2025;4:543-6. doi:10.1038/s44161-025-00642-0.

[59] Behradfar E, Nygren A, Vigmond EJ. The role of Purkinje-myocardial coupling during ventricular arrhythmia: A modeling study. PLoS ONE. 2014;9:e88000. doi:10.1371/journal.pone.0088000.

[60] Vigmond EJ, Clements C. Construction of a computer model to investigate sawtooth effects in the Purkinje system. IEEE Transactions on Biomedical Engineering. 2007;54:389-99. doi:10.1109/TBME.2006.888817.

[61] Garcia-Bustos V, Sebastian R, Izquierdo M, Rios-Navarro C, Bodí V, Chorro FJ, et al. Changes in the spatial distribution of the Purkinje network after acute myocardial infarction in the pig. PLoS ONE. 2019;14:e0212096. doi:10.1371/journal.pone.0212096.

[62] Veenstra RD, Joyner RW, Rawling DA. Purkinje and ventricular activation sequences of canine papillary muscle. Effects of quinidine and calcium on the Purkinje-ventricular conduction delay. Circulation Research. 1984;54:500-15. doi:10.1161/01.RES.54.5.500.

[63] Berenfeld O, Jalife J. Purkinje-muscle reentry as a mechanism of polymorphic ventricular arrhythmias in a 3-dimensional model of the ventricles. Circulation Research. 1998;82:1063-77. doi:10.1161/01.RES.82.10.1063.

[64] Ronaldson-Bouchard K, Ma SP, Yeager K, Chen T, Song L, Sirabella D, et al. Advanced maturation of human cardiac tissue grown from pluripotent stem cells. Nature. 2018;556:239-43. doi:10.1038/s41586-018-0016-3.

[65] Ma J, Guo L, Fiene SJ, Anson BD, Thomson JA, Kamp TJ, et al. High purity human-induced pluripotent stem cell-derived cardiomyocytes: Electrophysiological properties of action potentials and ionic currents. American Journal of Physiology-Heart and Circulatory Physiology. 2011;301:H2006-17. doi:10.1152/ajpheart.00694.2011.

[66] Sánchez-Quintana D, Yen Ho S. Anatomía de los nodos cardíacos y del sistema de conducción específico auriculoventricular. Revista Española de Cardiología. 2003;56:1085-92. doi:10.1157/13054255.

[67] Sebastian R, Zimmerman V, Romero D, Sánchez-Quintana D, Frangi AF. Characterization and modeling of the peripheral cardiac conduction system. IEEE Transactions on Medical Imaging. 2013;32:11. doi:10.1109/TMI.2012.2221474.

[68] Frantz S, Hundertmark MJ, Schulz-Menger J, Bengel FM, Bauersachs J. Left ventricular remodelling post-myocardial infarction: Pathophysiology, imaging, and novel therapies. European Heart Journal. 2022;43:2549-61. doi:10.1093/eurheartj/ehac223.
